# Anti-adipogenic effect of *Malva parviflora* on 3T3-L1 adipocytes

**DOI:** 10.1371/journal.pone.0306903

**Published:** 2024-08-08

**Authors:** Marisol Méndez-Martínez, Alejandro Zamilpa, Miguel A. Zavala-Sánchez, Julio C. Almanza-Pérez, J. Enrique Jiménez-Ferrer, Maribel Herrera-Ruiz, Manasés González-Cortázar, Jaquelynne Cervantes-Torres, Gladis Fragoso, Gabriela Rosas-Salgado

**Affiliations:** 1 División de Ciencias Biológicas y de la Salud, Departamento de Sistemas Biológicos, Universidad Autónoma Metropolitana, Mexico City, Mexico; 2 Facultad de Medicina, Universidad Autónoma del Estado de Morelos, Cuernavaca, Morelos, Mexico; 3 Centro de Investigación Biomédica del Sur, Instituto Mexicano del Seguro Social, Xochitepec, Morelos, Mexico; 4 Laboratorio de Farmacología, División de C.B.S., Departamento de Ciencias de la Salud, Unidad Iztapalapa, Universidad Autónoma Metropolitana, Mexico City, Mexico; 5 Departamento de Inmunología, Instituto de Investigaciones Biomédicas, Universidad Nacional Autónoma de México, Mexico City, Mexico; Foshan University, CHINA

## Abstract

*Malva parviflora* has shown anti-inflammatory, antihypertensive, antihyperlipidemic, and hypoglycemic effects. This study is aimed to evaluate the anti-adipogenic effect of *M*. *parviflora* on 3T3-L1 adipocytes. Fibroblast differentiation was induced either in the absence or presence of *M*. *parviflora* fractions (F3, F4, F7, F12, F13, F17, F18 and F19) for 4 days; F17 and 18 were the most effective fractions in reducing intracellular lipid accumulation (by 25.6% and 23.1%, respectively). EC_50_ of F17 and F18 (14 μg/mL and 17 μg/mL, respectively) were used to evaluate their anti adipogenic effect. After 10 days of inducing differentiation in the absence or presence of the extracts at the EC_50_ of F17 and F18, lipid accumulation, the concentration of interleukin 6 (IL-6) were measured in the culture medium; the presence of PPAR-γ, AKT, and p-AKT was also determined. In differentiated adipocytes (C2), F17 maintained intracellular lipid concentration at levels comparable to metformin, while decreasing PPAR-γ and increasing p-AKT presence; it also prevented IL-6 expression. F17 consists of alanine, valine, phenylalanine, and proline. On the other hand, F18 reduced intracellular lipid concentrations, prevented the increase of PPAR-γ and p-AKT, and maintained IL-6 expression at similar levels as metformin. F18 is mainly constituted by alanine, valine, proline, and sucrose. In conclusion, *M*. *parviflora* fractions (F17 and F18) control the process of adipogenesis, lipogenesis, and cellular dysfunction.

## 1. Introduction

Obesity is a major public health challenge in the world due to its magnitude, rapid increase, and negative impact on the health of the population that suffers from it, reaching epidemic proportions worldwide. Because of this, the World Health Organization (WHO) has named it the epidemic of the 21st century [1, 2].

Obesity is characterized by an increase in fat mass due to physical inactivity and a diet rich in fat and calories [3]. This leads to adipose tissue cells, adipocytes, increasing in mass (hypertrophy) and number (hyperplasia). Both processes are the result of adipocyte multiplication through processes such as adipogenesis, increased accumulation of triglycerides in the cytoplasm, and expression of lipogenic enzymes and adipokines, such as IL-6, which are associated with the development of insulin resistance [4].

Treatments to regulate adipocyte dysfunction are available, such as metformin, often used as an antihyperglycemic, insulin sensitizer, lipolysis regulator, and aids in body weight maintenance [5]. Thiazolidinediones like pioglitazone increase insulin sensitivity and regulate lipid metabolism, while activating transcription factors necessary for the development of the adipogenesis process, differentiating fibroblasts into adipocytes [6].

During adipogenesis there is 1) proliferation and differentiation of fibroblasts to preadipocytes, and 2) oxidation and synthesis of fatty acids (lipogenesis). Adipogenesis is controlled by molecular transcription and adipogenic factors; among the former, peroxisome proliferator-activated receptor γ (PPAR-γ), which is expressed in greater amounts in adipose tissue, is regarded as the main factor regulating adipogenesis and lipogenesis. The action of insulin during adipogenesis is related to the activation of the serine/threonine kinase (AKT) pathway, which phosphorylates and regulates a wide variety of substrates involved in GLUT4 translocation, thus increasing glucose consumption; it also promotes glycogen synthesis by phosphorylating glycogen synthase (GS) kinase and disinhibiting GS; additionally, it phosphorylates the transcription factor FOXO1, decreasing glucose synthesis, and increases lipid synthesis by activating the mTOR complex, which is linked to lipogenesis [7–9].

*Malva parviflora* (Malvaceae) is an herbaceous plant native to Europe, North Africa, and Asia, brought to America by European settlers [10]. It has been used in traditional medicine to treat fever, stomach pain, edema, and the flu [11]. It has shown antioxidant, anti-inflammatory, hypoglycemic, antihypertensive, and neuroprotective properties *in vivo*. These effects could be due to the presence of flavonoids, phenolic compounds, sterols, and fatty acids, which have been detected in phytochemical studies of the plant [12–15]. In models of obesity in which animals were maintained under obesogenic conditions, the use of *M*. *parviflora* prevented the change in body mass, even though the hypercaloric diet persisted [15]. This result was striking, because it is unknown whether the plant exerts a role in adipogenesis, so the effect of *M*. *parviflora* on adipogenesis in 3T3-L1 adipocytes is evaluated herein.

In this study, the effect of *M*. *parviflora* fractions, obtained from methanolic extract, was assessed on adipogenesis by measuring lipid accumulation. The EC_50_ of F17 and F18, the effective fractions, was determined, and it was tested whether the anti-adipogenic effect was due to a decrease in the levels of PPAR-γ, and phosphorylated AKT (p-AKT). IL-6 production was used as a marker of cell dysfunction. The phytochemical features of both fractions were determined.

## 2. Material and methods

### 2.1 Plant material and fractionation of *Malva parviflora*

The aerial parts of *M*. *parviflora* were collected in Ozumba de Alzate, State of Mexico, Mexico. One specimen was identified by Margarita Avilés and Macrina Fuentes, with reference voucher 2088 in the herbarium of the ethnobotanical garden of the Museo de Medicina Tradicional y Herbolaria, INAH, in Cuernavaca, Morelos. Leaves and flowers were dried at room temperature for 2 weeks. The dried material was ground in a grinder (Pulvex), obtaining particles of approximately 5 mm in diameter.

Dried, ground material (2 kg) was successively extracted with 3 solvents of ascending polarity. It was first macerated with 10 L dichloromethane for 24 h. After filtration, the extract was concentrated to dryness by reduced-pressure distillation in a rotary evaporator (Laborota 4000, Heidolph, Schwabach, Germany). The residual plant material was subsequently extracted with 10 L of acetone by maceration for 24 h, yielding an acetone extract, and again the dried plant was extracted with 10 L of methanol yielding a methanolic extract. The solvents were removed by rotaevaporation.

### 2.2 Chromatographic fractionation of methanolic extract

One hundred grams of the methanolic extract were weighed, adsorbed on the same amount of silica gel 60, and separated on a glass column previously packed with silica gel 60 (3 × 60 cm, 500 g, 70–203 mesh, Merck, Rahway, NJ). A dichloromethane/methanol gradient system was used as the mobile phase, collecting 100-mL fractions for a total of 19 fractions. The most abundant fractions (F3, F4, F7, F12, F13, F17, F18 and F19) were selected for further pharmacological evaluation.

### 2.3 Chemical purification of fractions F17 and F18

To identify the compounds found in the effective fractions of the methanolic extract of *M*. *parviflora* (F17 and F18), 1 gram of each fraction was passed through an open chromatographic column previously packed with 50 g of silica gel 60 (RP-18), in triplicate. The water:acetonitrile gradient system was used as the mobile phase, starting with 100% water, increasing the polarity in steps of 10% until reaching 100% acetonitrile, and collecting 10-mL samples. Fifty-five samples were obtained from each fraction. Fractions 1 to 5 (50 mg) of F17 were mixed for a reverse phase purification (RP-C18) where proline (**3**) was isolated (spectra in supplementary data (S2-S5 Fig in S1 File)) and also it was found in the mixture of the compound’s alanine (**1**), valine (**2**), proline (**3)**, phenylalanine (**4**) and the sugar know as sucrose (**5**) (spectra in supplementary data (S7-S9 Fig in S1 File)). In the other hand, in fraction F18 (4 mg), a mixture of **1**, **2**, **3**, and **5** was identified (supplementary data (S10-S17 Fig in S1 File)).

### 2.4 Acetylation, purification and identification of sucrose

The pools of each fraction (21.3 mg of F17 and 26.8 mg of F18) were independently acetylated with pyridine and acetic anhydride in a 1:3 ratio by incubating for 1 hour under heat and constant stirring; the reaction was stopped by adding ice, and subsequently ethyl acetate was added. The ethyl acetate (4.6 mg for F17aceto and 7.7 mg for F18aceto) was concentrate and purified through a normal-phase column (silica gel 60, 10 g); the dichloromethane:methanol gradient system was used as the mobile phase, starting with 100% dichloromethane and increasing the polarity in 2% steps, until reaching 92% dichloromethane. A total of 25 10-mL samples were obtained. The same procedure was carried out for both fractions. Samples 1 to 5 of each fraction were combined and sucrose was identified in F18 aceto (2 mg) was identified by ^1^H and ^13^C NMR (spectral in supplementary data (S18, S19 Fig in S1 File)).

### 2.5 Chemical characterization of active fractions

All spectra were recorded at 298°K on a Bruker Ascend 400 MHz NMR spectrometer, operated at 400 MHz for ^1^H-NMR and 100 MHz for ^13^C-NMR, using CD_3_OD (solvent used) for integral fractions F17 and F18. On the other hand, CDCI3 (solvent) was used to detect the sugar present in F18acet. Chemical shifts were report in ppm relative to Tetramethylsilane (TMS). The 2D spectra (COSY, HSQC, HMBC) were acquired only when necessary. For data analysis, CD_3_OD and CDCl3 solvent signal suppression was conducted.

### 2.6 Mass spectrometry

To identify the chemical compounds responsible for the biological activity of F17 and F18, a mass spectrometry analysis was performed at the Centro de Investigación Biomedica del Sur (CIBIS, Xochitepec, Mexico). To do this, 5 mg of each fraction was diluted in 0.05% trifluoroacetic acid to have a final concentration of 5 mg/mL. The samples (F17, subfraction F17 and F18) were analyzed on a triple quadrupole TQD mass spectrometer (Waters) through a combined electrospray-CAPIZ-spray ion source. All samples were analyzed for ions in positive and negative mode.

### 2.7 Thin layer chromatography

To confirm the chemical composition of fractions F17 and F18 of the methanolic extract of *M*. *parviflora*, in addition to nuclear magnetic resonance (NMR) spectroscopic data, the fractions were compared with standard reference amino acids (alanine, proline, valine, and phenylalanine), a terpene (ursolic acid), and β-sitosterol glycoside. A mixture of ethyl acetate:methanol:water:glacial acetic acid (5:5:2:0.5 v/v/v/v) was used as the mobile phase. The plates were developed with ceric ammonium sulfate, vanillin-H_2_SO_4_, and ninhydrin, according to the manufacturer’s instructions.

### 2.8 Quantification of amino acids present in F17 and F18

To quantify the concentration of the amino acids, present in F17 and F18, 5 different concentrations (1, 0.5, 0.25, 0.125, 0.0625 mg/mL) of the amino acid standards alanine, valine, proline and phenylalanine were used, diluted in distilled water, while that 3 mg of each fraction was diluted in 1 mL of methanol obtaining a final concentration of 3 mg/mL. Subsequently, 10 μL of each sample was placed on normal phase chromatographic plates. The system used was ethyl acetate:methanol:water:glacial acetic acid (5:5:2:0.5). To visualize the compounds, the ninhydrin reagent (Merck) was used, following the manufacturer’s instructions. Subsequently, photographs were captured for digitization of the thin layers and the determination of the intensity patterns of the sample marks was carried out through the software ImageJ. The respective calibration curves were made, calculating the correlation coefficient (r) and the equation of the line. The concentration of amino acids in the fractions was calculated in mg/g of fraction. These evaluations were performed in triplicate.

### 2.9 Cell culture, differentiation, and treatment

Murine 3T3-L1 fibroblasts were purchased from American Type Culture Collection (ATCC CL-173; Rockville, MD) and cultured with high-glucose Dulbecco’s Modified Eagle’s Medium (DMEM, ATCC 30–2002) supplemented with 10% fetal bovine serum (FBS, Corning 35-022-CV, Corning, NY) and 20 μg/mL gentamicin (Gibco 15710–64, Grand Island, NY), and incubated at 37°C, 5% CO_2_, for 48 h. Then, fibroblasts were differentiated to preadipocytes by culturing a differentiation medium containing 1.0 μM dexamethasone (Sigma D1881, St. Louis, MO), 0.5 mM methyl-isobutyl-xanthine (MIBX, Sigma 15879), and 1.0 μg/mL insulin (Sigma 1882) in DMEM with 10% FBS (Gibco, 16000–069) plus the treatments described below (experimental day 0). Two days later (experimental day 2), the medium was replaced with a maintenance medium (DMEM with 10% FBS and 1.0 μg/mL insulin), plus treatments. The cells were maintained for 2 (pre-adipocyte) or 8 (adipocyte) further days with maintenance medium, according to the experimental procedure.

This study was approved by the investigation committee whit the No. 2024–158

### 2.10 Toxicity of *M*. *parviflora* fractions and pools evaluated by MTT

To assess whether *M*. *parviflora* fractions (F3, F4, F7, F12, F13, F17, F18, and F19) are toxic to cells, fibroblasts were exposed to 60 μg/mL of each treatment once differentiation was induced (days 0–2); the effect was continued on days 3 and 4 of culture, in maintenance medium; 1.5 × 10^3^ cells/well were seeded in 96-well plates. Undifferentiated fibroblasts (C1) and untreated differentiated fibroblasts (C2) were included as controls, as well as cells exposed to DMSO (60%) as a toxicity control, metformin (Met) (1 mM) as anti-lipogenic control [5], or pioglitazone (Pio) (20 μM) as adipogenic control [6]. Each treatment was applied in duplicate, and three replicates were performed.

Toxicity was assessed on experimental day 4. The culture medium was removed from the cells, and they were exposed to 10 μL of a solution of 3-(4,5-dimethylthiazol-2yl)-2,5-diphenyltetrazolium, 5 mg/mL (MTT, Sigma M2128), at 37°C, for 4 h. Then, 100 μL of SDS 10% HCl 0.01 N (1:1) were added to each well, and they were incubated for further 2 h at 37°C. Then, absorbance was read on a VERSAmax microplate reader (Molecular Devices, Sunnyvales, CA) at 570 nm. Cell viability was expressed as a percentage relative to C1.

### 2.11 Identification of anti-lipogenic pools or fractions

Cells were seeded in 24-well plates at a density of 3 × 10^4^ cells/well, as described above. Fibroblasts cultured in DMEM with 10% SFB with no differentiation inducers (C1) were included as controls, as well as differentiated, untreated fibroblasts (C2), or cells cultured in differentiation medium first and then in maintenance medium, with Pio or Met. Experimental treatments (60 μg/mL) were diluted first in differentiation medium (2 days) and then in maintenance medium (another 2 days). Each condition was evaluated in duplicate, with 3 replicates. The anti-lipogenic activity was estimated by the concentration of intracellularly accumulated lipids, calculated by Red-Oil-O staining.

A pilot experiment was carried out to know the concentration at which pioglitazone induced an increase in the concentration of lipids, for these two concentrations were used, one of 10 μM and the other of 20 μM (S21 Fig in S1 File).

### 2.12 Red-Oil-O staining

After 4 days of culture, the cells were carefully washed twice with phosphate buffer saline (PBS) and fixed with 10% formalin for 60 min. Upon formalin removal, 60% isopropanol was added to each well, and the plates were incubated for 5 min for cell permeabilization. The cells were then incubated with Red-Oil-O working solution (Sigma O 0625) for 10 min and washed once with distilled water and once with tap water. Intracellular lipids were extracted with 100% isopropanol. The plates were read at 500 nm in a VERSAmax microplate reader. The results were expressed as absorbance values. Each condition was assessed in duplicate, with three replicates. Adipocyte maturity on day 10 of culture was also assessed by this method.

### 2.13 EC_50_ determination for F17 and F18

Once the anti-lipogenic activity was identified in F17 and F18, the EC_50_ was calculated and used in further evaluations. Fibroblasts were seeded in 24-well plates as described above, with F17 and 18 at concentrations of 30, 60, 120, 240, or 480 μg/mL. Upon incubation, lipid accumulation was assessed by the Red-Oil-O technique, and EC_50_ and E_max_ were calculated by concentration-response curves.

### 2.14 Protein extract

For protein quantification, 3T3-L1 cells were seeded in 24-well plates. After 10 days of culture, the medium was removed, and the cells were washed with cold PBS. Then, they were incubated in RIPA lysis buffer (20 mM Tris, pH 7.4; 150 mM NaCl, 1 mM EDTA, pH 7.4; 0.5% Triton X-100, 0.1% SDS, 0.5% sodium deoxycholate) at 4°C, using a protease and phosphatase inhibitor cocktail (25 mM NaF, 1 mM NaPPi, 1 mM NaVPO_4_, 1 mM PMSF, 0.1 mg/mL pepstatin A, 0.1 mg/mL leupeptin, 0.1 mg/mL aprotinin, Sigma) for 15 min.

To extract nuclear proteins, the cells were incubated in Petri dishes (60 mm x 15 mm) at a density of 1.2 × 10^5^ cells under the conditions described above. After 10 days of culture, the cells were incubated at 4°C with 0.05 mM EDTA in PBS for 10 min. The cells were lifted from the Petri dish with a cell filter and centrifuged at 134 × *g* for 5 min at 4°C. The pellet was resuspended with lysis buffer A (10 mM HEPES, 10 mM KCl, 0.1 mM EDTA, 0.1 mM EGTA, 1 mM DTT), adding a cocktail of protease and phosphatase inhibitors, and incubated for 15 min on ice. Then, 10% Nonidet P40 solution was added, mixed for 10 s, and centrifuged at 15,600 × *g* for 1 min. The nuclear pellet was resuspended in nuclear lysis buffer (20 mM HEPES, 0.4 M NaCl, 1 mM EDTA, 1 mM EGTA, 1 mM DTT) in the presence of a phosphatase and protease inhibitor cocktail; then, it was mixed for 15 min at 4°C and centrifuged at 15,600 × *g* for 5 min. Protein concentration was measured by the Lowry method.

### 2.15 Western blot analysis

To assess the effect of the fractions on cell differentiation, the presence of PPAR-γ, AKT, and p-AKT was detected by Western blot. Equal protein amounts from each sample were placed on SDS-PAGE gels in 10% acrylamide and transferred onto a polyvinylidene difluoride (PVDF) membrane (Merck, Darmstadt, Germany). For AKT and p-AKT analysis, the membranes were blocked for 1 h with Tris Buffered saline buffer with Tween 20 0.055 (TBS-T) and 2% bovine serum albumin (BSA) or 5% milk. Blocking was performed with anti-AKT (Cell Signaling 9272S, Danvers, MA) and anti-p-AKT (Cell Signaling 9271S) antibodies. The bands were visualized with HRP-conjugated anti-rabbit secondary antibodies (Abcam, Cambridge, MA). Antibody binding was detected with the Super Signal West Dura Extended Duration Substrate solution. The bands were analyzed with the software ImageJ (National Institute of Health, Bethesda, MD), normalizing them with respect to anti-β-actin signals (Abcam ab8227). To analyze the results, a p-AKT/AKT index was calculated.

For PPAR-γ analysis, 10 μg of nuclear protein were placed on an SDS-PAGE gel in 10% acrylamide and transferred onto a PVDF membrane. The membrane was blocked for 1 h with a TBS-T buffer with 20% milk. The membranes were incubated overnight with anti-PPAR-γ antibody (Invitrogen MA5-14889, Carlsbad, CA) or anti-laminin B1-HRP antibody (Abcam). The bands were visualized with an anti-rabbit-HRP antibody. Antibody binding was detected with Super Signal West Dura Extended Duration Substrate solution. The bands were analyzed using the software ImageJ 1.8.0, using the laminin B1 signal (Abcam ab16048) for normalization.

### 2.16 Quantification of IL-6 in culture medium by sandwich ELISA

Once the mechanism of action of the anti-adipogenic effect of F17 and F18 was explored, both fractions were administered at their respective EC_50_ to evaluate their effect on the dysfunctional condition of the cell through kinetics of the expression of IL-6. Fibroblasts were seeded in 24-well plates under the conditions described above, although this time the culture was continued until day 10 in maintenance medium. Samples to measure IL-6 concentration were taken on days 4 and 10 of culture, and analysis was performed with the ELISA kit (OptEIATM BD, 555240, San Diego, CA), following the manufacturer’s instructions. Protein content was determined by the Bradford assay with a commercial kit (Sigma B6916), following the manufacturer’s instructions.

### 2.17 Statistical analysis

Data was collected in Excel (Microsoft, Redmond, WA). In toxicity evaluation the data are expressed as mean ± SEM. The data obtained from the other evaluations are presented as box-and-whisker plots. The software INSTAT 3 (GraphPad, La Jolla, CA) was used to perform uni- and multivariate analyses of the results. Differences were evaluated using ANOVA and the post-hoc Tukey-Kramer test. Differences were considered statistically significant for *p* ≤ 0.05 and *p < 0*.*01*.

## 3. Results

### 3.1 F17 and F18 prevent intracellular lipid accumulation

**Fig 1:**
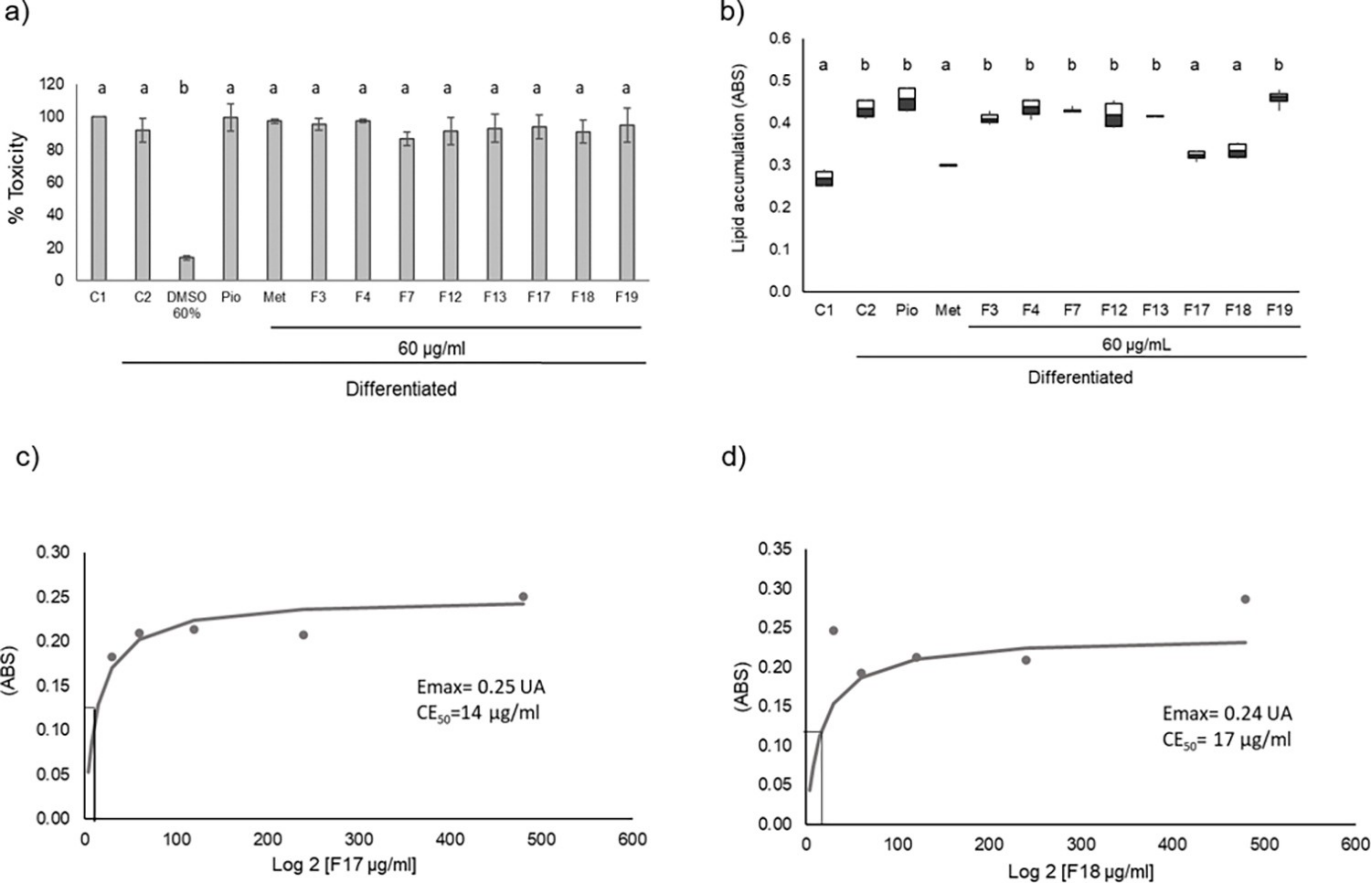
Effect of *M*. *parviflora* fractions on cell toxicity (a) and lipid accumulation (b) during the 4 days of differentiation of 3T3-L1 fibroblasts into preadipocytes. Concentration-response curve of F17 (c) and F18 (d) of lipid accumulation in the presence of the differentiation inducers plus 30, 60, 120, 240 or 480 μg/ml of the fractions, from which the maximum effect (E_max_) was calculated and the effective concentration 50 (EC_50_). Results of toxicity assays for both fractions are reported as mean ± SD; lipid accumulation is shown in box-and-whisker plots of three independent experiments and kinetics curve for calculated Emax and CE_50_. Literal differences represent statistical differences p≤ 0.001, compared to control undifferentiated cells (C1). C2: differentiated cells, Met: Metformin (10 mM), Pio: Pioglitazone (20 μM), F: Fraction of *M*. *parviflora*. Data were analyzed with the ANOVA and post-hoc Tukey-Kramer tests, n = 3.

To identify the extract fractions responsible for preventing intracellular lipid accumulation, the toxicity of these fractions on preadipocytes was evaluated. To this end, an MTT assay was performed after 4 days of incubation in the presence of the treatments plus differentiation inducers. Our results (Fig 1A) indicated that the fractions were not toxic to the cells, as they did not show significant differences with respect to the controls C1 and C2, while the cell death control (DMSO 60%) showed an 86% decrease in cell viability (*p < 0*.*001*). This result validates the use of these treatments and controls under the conditions used to determine the EC_50_.

Once the safety of the *M*. *parviflora* fractions was established, those that prevent intracellular lipid accumulation were identified by Red-Oil-O staining. Fibroblasts were cultured under the permanent effect of the different treatments, first in differentiation medium and then in maintenance medium. The results of the pilot experiment with respect to the effective concentration of pio, showed that the concentration of 20 μM increased significantly by 61% more than the C1 cells, while those that were maintained at concentration 10 μM increased by 5% and did not present significant differences with respect to C1 cells (S20 Fig in S1 File).

As shown in Fig 1B, of the different fractions evaluated, only F17 and F18 significantly decreased intracellular lipid accumulation (25.6% and 23.1%, respectively, *p < 0*.*001*) with respect to C2 (cells treated with differentiation inducers as insulin, isobutyl-methyl-xanthine, and dexamethasone), with a similar effect to metformin (31%). These fractions, like Met, kept intracellular lipid concentrations at the level of undifferentiated fibroblasts (C1).

This result indicates that, of all plant-derived fractions, only F17 and F18 prevented intracellular lipid accumulation and thus adipogenesis, potentially preventing transformation from fibroblasts into pre-adipocytes and then into adipocytes. Meanwhile, cells treated with the fractions F3, F4, F7, F12, F13, and F19 showed no significant differences with respect to the control group (Pio) nor to the C2 group.

To evaluate the anti-adipogenic and anti-lipogenic effects of F17 and F18, the EC_50_ was calculated for each fraction during the preadipocyte state (first 4 days of culture). Five different concentrations of each fraction were added, and their effect on Red-Oil-O accumulation was measured in the presence of differentiation inducers.

As shown in Fig 1C and 1D, the fractions have a concentration-dependent effect, where F17 has an E_max_ = 0.25 AU and an EC_50_ = 14 μg/mL (panel c). While the F18 has an E_max_ = 0.24 AU and an EC_50_ = 17 μg/mL (panel d).

### 3.2 F18 prevents lipid accumulation and control adipogenesis through PPAR-γ within the cell

Once EC_50_ was determined for both fractions, their effect on adipocyte maturity was assessed by measuring lipid accumulation after 10 days of incubation by Red-Oil-O staining.

**Fig 2:**
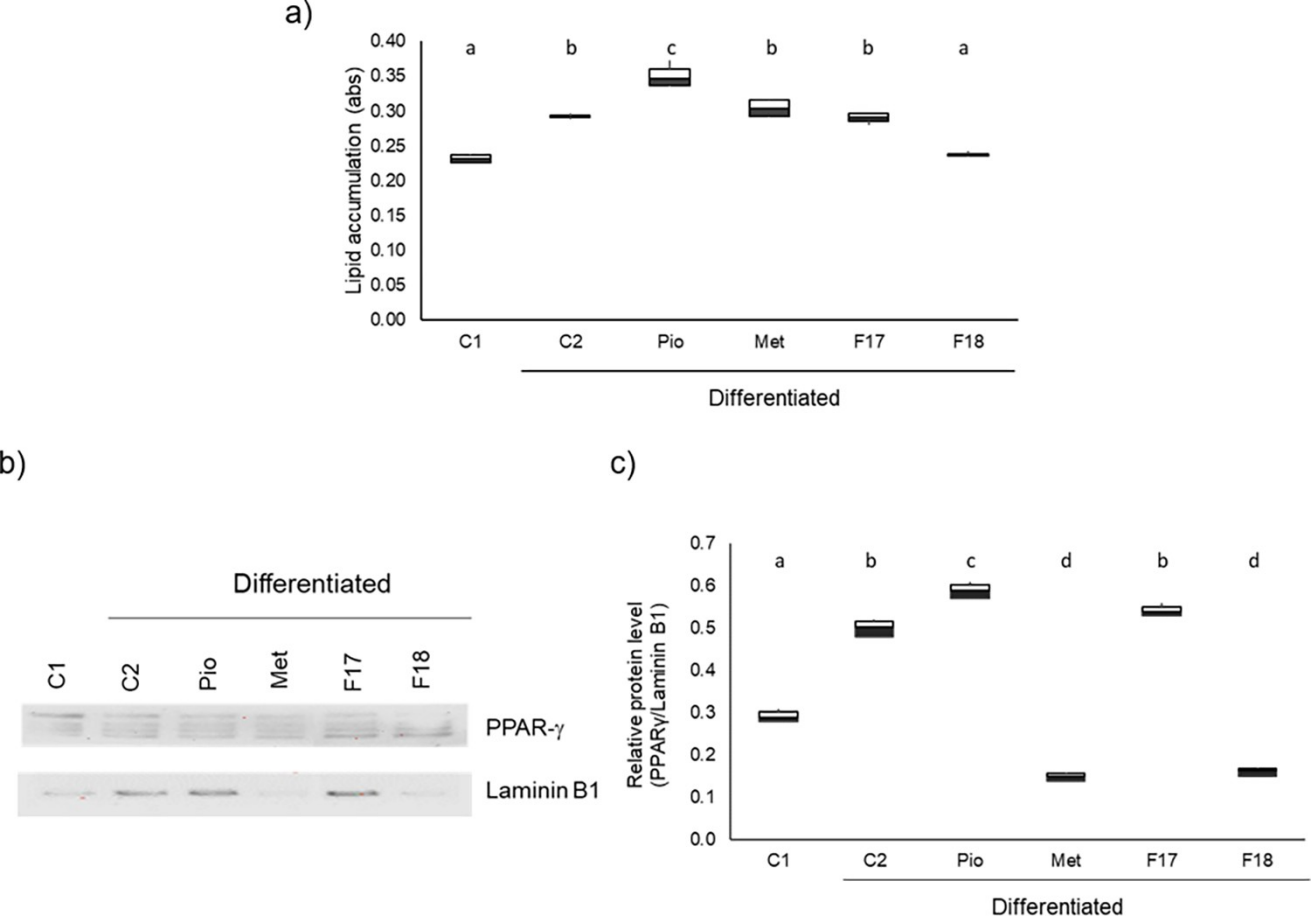
Effect of the fractions on lipid accumulation and PPAR-γ levels after 10 days of incubation. a) lipid accumulation, b) Western blot analysis of PPAR-γ band used for quantification. c) box-and-whisker plots represent the band intensities from western blot PPAR-γ adjusted by Laminin B1. Cells were differentiated (days 0–2) and maintained (days 3–10) in the presence or absence of the EC_50_ of F17 (14 μg/mL) or F18 (17 μg/mL) or Pio (20 μM) or Met (10 mM). Results are shown in box-and-whisker plots of three independent experiments. Different literals indicate significant statistical differences p≤ 0.001, compared to control cells (C1). C2: differentiated cells, Met: Metformin, Pio: Pioglitazone, F: Fraction of *M*. *parviflora*. Data were analyzed with the ANOVA and post-hoc Tukey-Kramer tests, n = 3.

As shown in Fig 2A, after 10 days of incubation, lipid intracellular concentration increased in all groups with respect to fibroblasts (C1) except in F18-treated cells, even in the presence of inducers. In mature adipocytes (group C2), an increase of 28.8% (*p* < 0.001) was observed; in cells treated with Pio, it was 51.1% (*p* < 0.001); in those treated with Met, it was 31.2%, similar to F17, which increased lipid levels by 25%. Only F18 showed results similar to group C1 (*p > 0*.*05*), because it only increased intracellular lipid concentration by 2.5%.

PPAR-γ is a transcription factor, which is required for transforming fibroblasts into preadipocytes [4]. To verify whether the fractions prevented an increase in the expression of this factor and to relate the effect of the fractions on intracellular lipid accumulation and adipocyte maturity after 10 days of incubation, the presence of PPAR-γ in cell nuclei was assessed by Western blot. As shown in panels b and c, under the presence of the inducers, C2, Pio, and F17 increased PPAR-γ concentration by 71% (*p < 0*.*001*), 100% (*p < 0*.*001*), and 84.9% (*p < 0*.*001*), respectively, with regard to undifferentiated fibroblasts (C1). On the other hand, Met and F18 decreased such levels (*p < 0*.*001*) by 48.5% and 44.7%, respectively.

This result indicates that, of the two fractions evaluated, F18 was the most efficient in controlling the adipogenesis process by decreasing the accumulation of cytoplasmic lipids and PPAR-γ levels, similar to Met.

### 3.3 F17 upregulated AKT phosphorylation while F18 downregulated phosphorylation

ATK is a kinase that, when activated by phosphorylation, initiates the process of maturation of adipocytes through lipogenesis [8].

**Fig 3:**
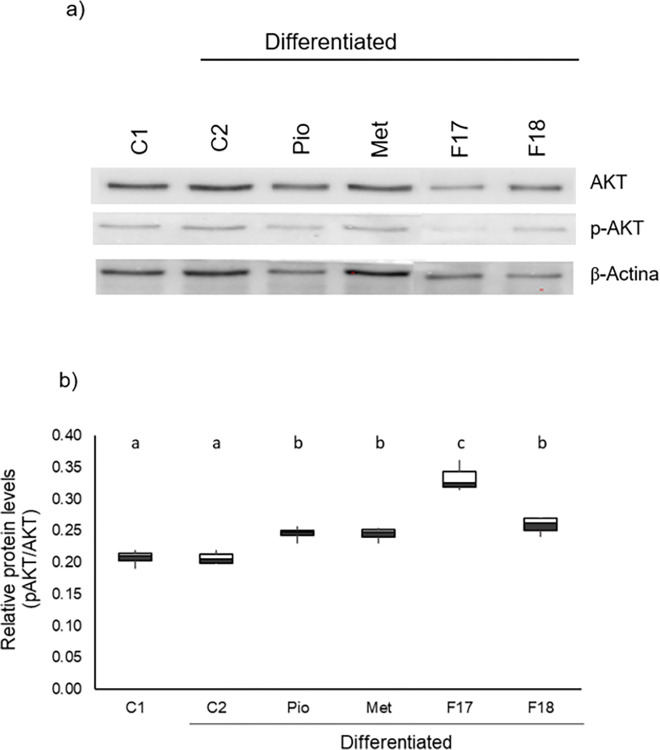
Effect of fractions F17 and F18 of *M*. *parviflora* extract on p-AKT/AKT levels during differentiation of 3T3L-1 fibroblasts at day 10 of differentiation. a) Western blot of AKT, p-AKT and β-actin used for quantification. b) Box-and-whisker plots of band intensity in Western blot for p-AKT/AKT. Cells were differentiated (days 0–2) and maintained (days 3–10) in the presence or absence of the EC_50_ of F17 (14 μg/mL), F18 (17 μg/mL), Pio (20 μM) or Met (10 mM). The plots show the result of three independent experiments; different letters indicate statistically significant differences, *p* ≤ 0.001, with respect to undifferentiated (C1) and differentiated (C2) cells. Results were analyzed with ANOVA and Tukey-Kramer post-hoc tests, n = 3.

To relate the effect of both fractions with lipid accumulation, the presence of AKT and p-AKT in the cytoplasm of cells under the different treatments were identified by Western blot analysis. The blots (panel a) and the plots resulting from the p-AKT/AKT index calculated (panels b) are shown in Fig 3.

No significant differences were observed in p-AKT/AKT levels between fibroblasts (C1) and differentiated adipocytes (C2) (*p > 0*.*05*) (Fig 3B). Meanwhile, Pio, Met, F17, and F18 increased the phosphorylate AKT with respect to C1 and C2. F17 increased phosphorylation by approximately 61%, while Pio increased it by 17%, Met by 18%, and F18 by 25% with respect to both controls.

These results indicate that F17 increases AKT phosphorylation, while F18 modulates this activation, which may be related to a modulation of lipid accumulation and adipocyte maturation.

### 3.4 F17 and F18 regulate IL-6 production

**Fig 4:**
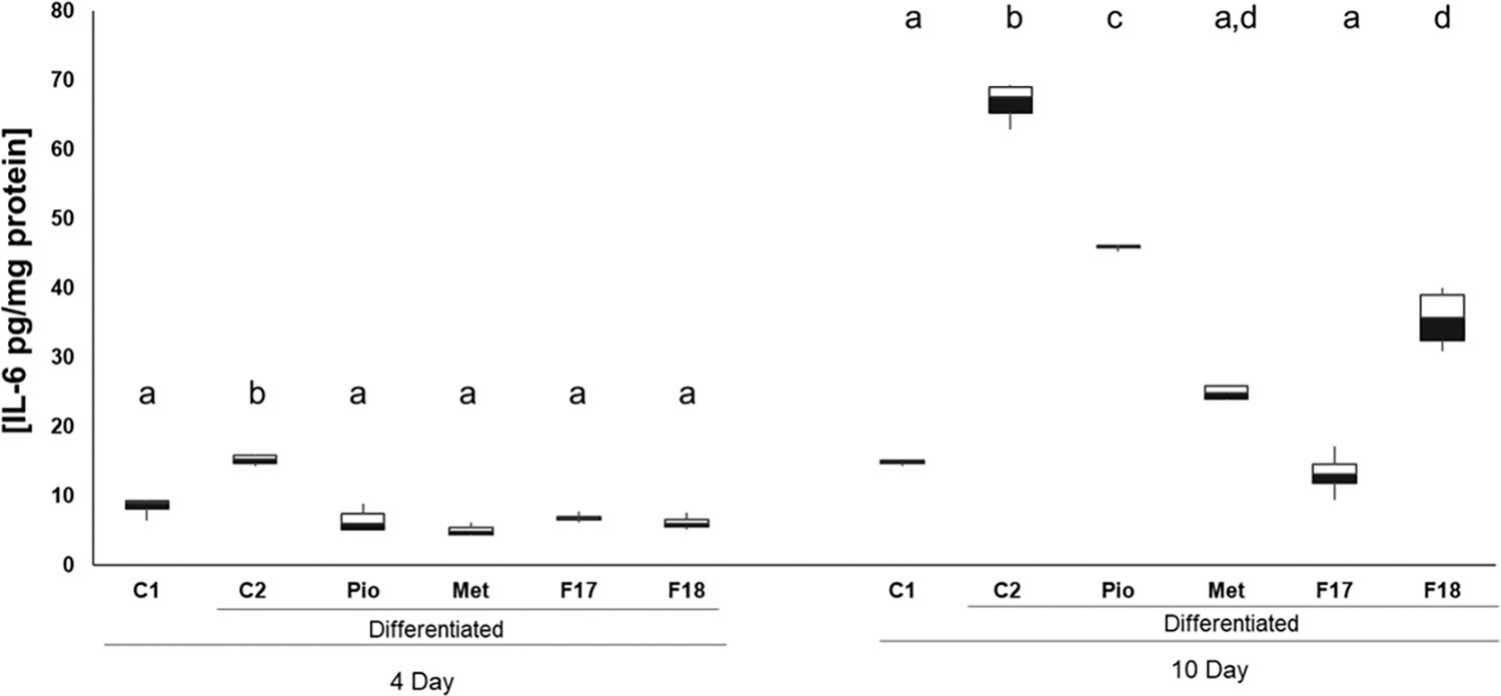
Effect of F17 and F18 fractions on IL-6 secretion. Cell culture medium was obtained at 4 and 10 days of culture Results are shown in box-and-whisker plots of three independent experiments; different literals indicate statistically significant differences, *p* ≤ 0.001, compared with the undifferentiated control (C1) at each time evaluated, C2: differentiated cells, Met: Metformin (10 mM), Pio: Pioglitazone (20 μM), F: Fraction of *M*. *parviflora* extract. Data were analyzed with the ANOVA and post-hoc Tukey-Kramer tests, n = 3.

Adipose tissue is considered an endocrine organ, which is capable of secreting different adipokines, among which are proinflammatory cytokines such as IL-6, which is associated with a dysfunctional state of adipocytes [16]. Once the mechanism of action of F17 and F18 on adipogenesis and adipocyte maturation was determined, their effect on IL-6 production (as a marker of adipocyte dysfunction) was evaluated on days 4 (stated pre-adipocyte) and 10 (stated adipocyte) of culture by sandwich ELISA. As shown in Fig 4, only the cells in group C2 showed increased concentrations of this cytokine on day 4 of culture compared with C1 (81%, *p* < 0.001), while no significant differences were observed in the experimental groups with respect to the untreated control cells (C1). After 10 days of culture, when adipocytes were already differentiated and dysfunctional [17], IL-6 concentrations increased by 298% (*p* < 0.001) with respect to untreated cells (Fig 4). Treatment with Pio and Met reduced the levels of this cytokine by 31.3% (*p* < 0.001) and 68.2% (*p* < 0.001), respectively, with respect to C2. Meanwhile, F18 reduced IL-6 levels by 46.5% (*p* < 0.001), an effect similar to that of Met, while F17 decreased this parameter by 80.2% (*p* < 0.001), reverting it to the level of untreated cells.

### 3.5 F17 and F18 are mainly composed by nonpolar and aromatic amino acids

To elucidate the composition of the effective fractions of *M*. *parviflora*, a thin-layer chromatographic analysis was performed. On developing the spots with ninhydrin, the plates indicated the presence of compounds with an amino group, so an acetylation reaction was performed in a pool with the first five samples of each fraction. In addition, NMR and mass spectrometry (Positive mode, (S1-S17 Fig in S1 File)) spectra showed the presence of the four amino acids alanine, valine, proline, and phenylalanine for F17, and alanine, proline, and valine for F18. The spectroscopic data are consistent with what was reported [18–21]. Thus, the presence of a sugar was also found, which through acetylation and NMR was identified as sucrose (Supplementary data S18, S19 Figs in S1 File).

#### Valine

^1^H NMR (400 MHz, CD_3_OD) δ 1.47 (1H, d J = 7.25 Hz, H-1), 3.99 (1H, d J = 2.61 Hz, H-2), 2.12 (1H, q, H-3), 1.02 (3H, d J = 6.97 Hz, H-4), 1.06 (3H, d J = 7.06 Hz, H-5). ^13^C NMR (100 MHz, CD_3_OD) δ 173.77 (COOH, C-1), 61.67 (CH, C-2), 30.4 (CH, C-3), 16.44 (CH_3_, C-5), 17.29 (CH_3,_ C-4).

#### Alanine

^1^H NMR (400 MHz, CD_3_OD) δ 1.46 (1H, d J = 7.16 Hz, H-1), 3.83 (1H, s, H-2), 1.32 (1H, d J = 6.49 Hz, H-3). ^13^C NMR (100 MHz, CD_3_OD) δ 174.06 (COOH, C-1), 51.78 (CH, C-2), 19.14 (CH_3_, C-3).

#### Proline

^1^H NMR (400 MHz, CD_3_OD) δ 4.00 (1H, dd J = 8.7–6.3 Hz, H-2), 2.3–2.2 (2H, m, H-3a), 2.11 (2H, dd J = 13.2 Hz, H-3b), 1.96–2.01 (2H, m, H-4a), 1.9–1.98 (2H, m, H-4b), 3.39 (2H, dt J = 11.4–6.9 Hz, H-5a), 3.25 (2H, dt J = 11.4–7.3 Hz, H-5b). ^13^C NMR (100 MHz, CD_3_OD) δ, 62.01 (CH, C-2), 30.42 (CH_2_, C-3), 25.13 (CH_2_, C-4), 46.98 (CH_2,_ C-5), 174.18 (COOH, C-6).

#### Phenylalanine

**Fig 5:**
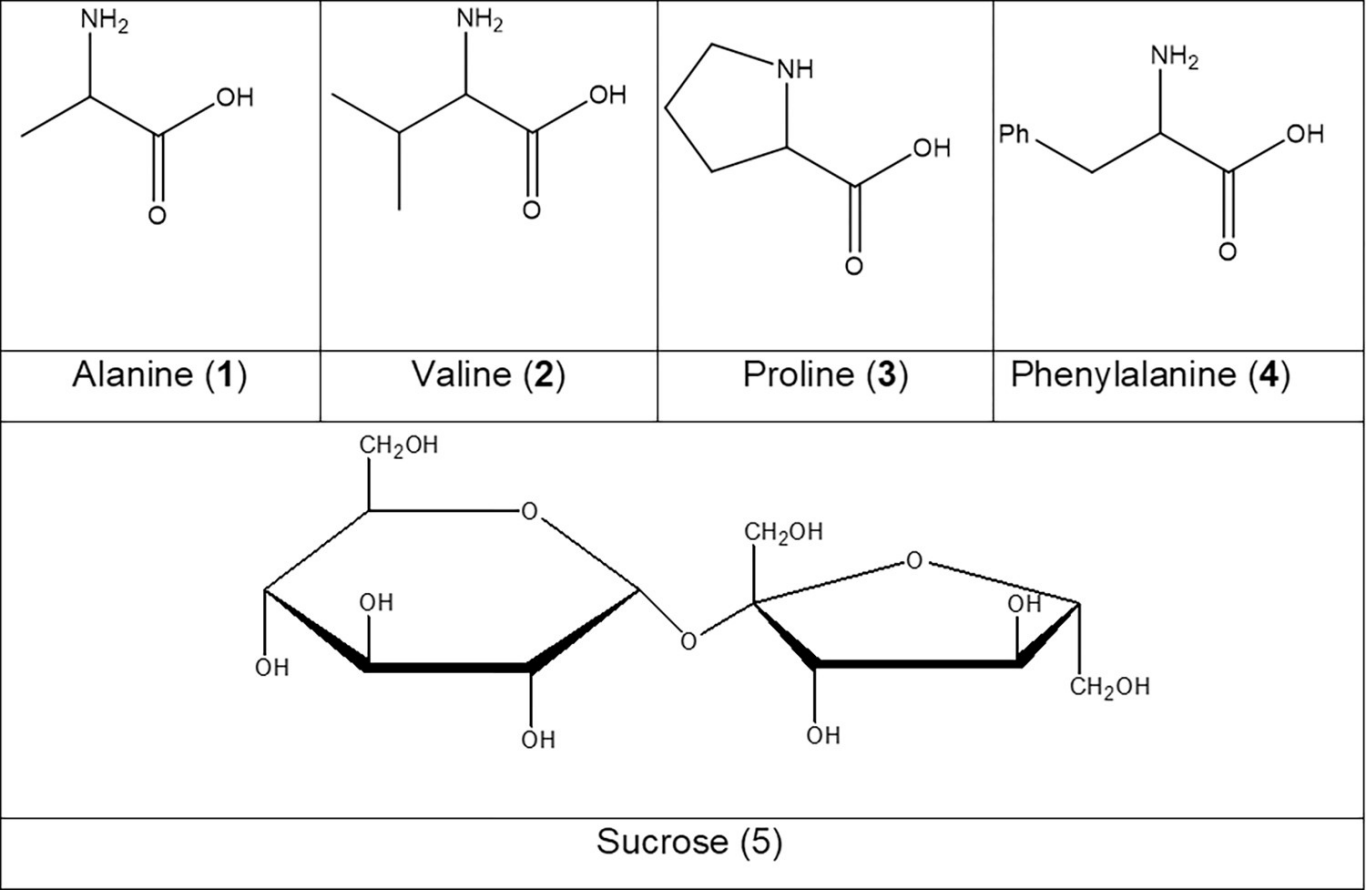
Structural molecule of alanine (1), valine (2), proline (3) phenylalanine (4) and sucrose (5).

^1^H NMR (400 MHz, CD_3_OD) δ 4.1 (1H, dd J = 8.9,4.3 Hz, H-2), 3.3 (2H, dd J = 14.5, 4.2 Hz, H-3a), 3.14 (2H, dd J = 14.5, 8.9 HZ, H-3b), 7.49 (2H, d J = 7.0 Hz, H-5-9), 7.35 (2H, d J = 7.6 Hz, H-6-8), 7.3 (1H, d, J = 6.9 Hz, H-7). ^13^C NMR (100 MHz, CD_3_OD) δ 176.68 (COOH, C-1), 57.7 (CH, C-2), 37.8 (CH_2_, C-3), 136.83 (C-4), 130.66 (CH_2_, C-5-9), 130.17 (CH_2,_ C-6-8), 128.5 (CH, C-7). The structural formula of both compounds is shown in Fig 5A–5D.

Thin layer chromatographic plates are shown in the S20 Fig in S1 File. The ninhydrin-developed plates, showing the presence of the aminoacids alanine, valine, phenylalanine, and proline in the F17 fraction, and of alanine, valine, and proline in the F18 fraction are shown in S20E and S20F Fig in S1 File., respectively. These findings are consistent with results obtained in NMR and mass spectrometry assays (S1–S19 Figs in S1 File). The plates developed with vanillin-H_2_SO_4_ are shown in S20A and S20B Fig in S1 File. As shown, no chemical compounds that react with this oxidizing mixture were found in the extracts. Finally, small spots of compounds with lower polarity than amino acids were observed in the plates developed with ceric ammonium sulfate; however, the concentration of these potential constituents is very low, as the intensity of other possible compounds in the NMR assays was not significant.

The concentrations of the amino acids present in the fractions are shown in Table 1. Where it is observed that F17 has a higher concentration of amino acids than F18. Based on the concentration of the amino acids measured in the fractions, it can be estimated that in 14 μg/mL of F17 contain 2.6 μg of proline, 1.27 μg of alanine, 0.82 μg of valine, and 1.78 μg of phenylalanine. On the other hand, 17 μg/mL of F18 contain 3.26 μg of proline, 1.46 μg of Alanine, and 0.16 μg of Valine.

**Table 1:** Concentration of amino acids present in F17 and F18.

Fraction	Ion mode	Positive Ion (m/z)	Linearity	Linear equation	Concentration (mg/g)
F17	Proline	116.06	0.9686	y = 50635x+11566	191.8
F17	Alanine	90.12	0.9576	y = 6235.1x+8989.5	91.01
F17	Valine	118.10	0.9769	y = 4643.8x+9894.6	58.63
F17	Phenylalanine	165.19	0.9649	y = 4518.3x+9544	126.95
F18	Proline	116.05	0.9187	y = 2321.7x+11582	191.94
F18	Alanine	118.07	0.9683	y = 5658.2x+9899.5	85.86
F18	Valine	166.08	0.9879	y = 5792.9x+8859.7	9.3

## 4. Discussion

This work lays the groundwork to study the mode of action of *M*. *parviflora* on adipogenesis in 3T3-L1 fibroblasts, aiming to develop a natural product that helps to control obesity and, thus, prevent associated diseases such as diabetes, cardiovascular diseases, malignant neoplasms, and osteoporosis [22], with the high cost to society in economic and mortality terms [23]. 3T3-L1 fibroblasts are widely used in studies on adipogenesis and energy metabolism, because in the presence of 1.0 μM dexamethasone, 0.5 mM methyl-isobutyl-xanthine, or 1.0 μg/mL insulin they can differentiate into mature adipocytes, with specific physiological and morphological characteristics, allowing exploration of the mechanisms underlying obesity and its possible treatment [24].

Previous work suggested that *M*. *parviflora* was able to prevent body weight gain in mice raised under obesogenic conditions [15]. To further study the mode of action of the plant, a methanolic extract of its aerial parts was fractioned; obtaining a total of 19 fractions. Interestingly, and despite the presence of inducers of differentiation to preadipocytes, F17 and F18 were the only fractions to show anti-lipogenic activity. Based on this capacity, EC_50_ was calculated for both fractions, with values of 14 μg/mL and 17 μg/mL, respectively. Interestingly, the administered concentration of both fractions was as effective as that of Met (which was administered at a much higher dose, about 1160 μg/mL) in modifying the parameters evaluated in this work. This may be because Met is a pure chemical species, whereas both fractions consist mainly of alanine, valine, and proline, with the inclusion of phenylalanine for F17, along with other minor constituents, like sucrose. This could allow them to act on several targets, preventing adipocyte maturation, intracellular lipid accumulation, and the establishment of a dysfunctional state.

Even though both fractions were effective to control adipogenesis, our results suggest that they act by different mechanisms, involving the PPAR-γ and p-AKT pathways. As previously reported, PPAR-γ is the main transcription factor for the initiation of adipogenesis. This transcription factor is required for the transformation of a fibroblast into an adipocyte, which is related to lipid intracellular accumulation. F18 was as efficient as Met in maintaining the levels of this transcription factor and lipids accumulation at similar values to those of non-differentiated fibroblasts despite the presence of inducers; this could indicate that, like Met, this fraction could activate the AMP-activated protein kinase (AMPK), thereby increasing the expression of mitochondrial respiratory chain proteins, favoring oxidative phosphorylation and thus reducing lipid droplet content in adipocytes [25]. While F17 did not reduce PPAR-γ levels, which remain the same as with Pio (20 μM), included as a positive control of differentiated cells [6]. On the other hand, the F18 plant-derived fraction was efficient in reducing p-AKT levels. p-AKT levels may indicate an insulin-induced adipogenic condition. Upon binding to insulin receptor (IR), insulin induces the phosphorylation of IRS, activating phosphoinositol-3 kinase (PI3K) pathway to activate AKT by 3-phosphoinositide-dependent protein kinase-1 (PDK-1). p-AKT increases lipid synthesis by activating the mTOR complex [7]. As mentioned above, Met prevents AKT activation by inhibiting mTOR [26]. A similar mechanism could also explain the effect of the F18 fraction.

On the other hand, adipocyte dysfunction also influences the production and accumulation of fat into the cell [27]. Both conditions were induced in fibroblasts cultured in differentiation medium [28]. Dysfunction is linked to adipogenesis by the activation of AKT, as it acts as a kinase for the phosphorylation and degradation of inhibitory κB (IκB), thereby activating NFκB and thus the secretion of proinflammatory cytokines, such as IL-6 [29]. IL-6, an adipokine produced by dysfunctional adipocytes, induces insulin resistance [27, 30]. In this study, both F17 and F18 prevent IL-6 secretion similarly to Met, a drug that not only regulates lipogenesis, adipogenesis, and adipocyte function, but also reduces the expression of proinflammatory cytokines. Met has been reported to inhibit the NF-κB signaling pathway [31], with the ensuing decrease in the levels of inflammatory cytokines such as IL-6. The production of this cytokine was also reduced by both fractions, a finding that suggests that F17 and F18 could act on the NF-κB pathway.

In previous studies, aerial parts of *M*. *parviflora* have shown antioxidant, anti-inflammatory, and hypoglycemic properties under obesity conditions. These properties have been attributed to the presence of flavonoids, phenolic compounds, sterols, and fatty acids [11–15]. Noteworthy, in this study, we found that the major compounds in fractions F17 and F18 have an amino group; NMR analysis identified these compounds as four amino acids, valine, alanine, proline, and phenylalanine for F17 or alanine, valine, and proline for F18. Amino acids, in addition to being the building blocks of proteins and polypeptides, can regulate metabolic pathways crucial for maintenance, growth, reproduction, and immunity, and they have begun to be used to improve protein balance, reduce adiposity, and improve health [32]. Valine, which is found in similar quantities in both fractions, has been reported to participate in the control of lipogenesis; Halperin and Robinson [33] found that valine administration in rats reduces fatty acid synthesis by decreasing the intracellular concentration of pyruvate, which acts as a source of Nicotinamide adenine dinucleotide phosphate (NADPH) and glycerol-3 phosphate. However, no reports have been published on its role in the regulation of PPAR-γ and IL-6.

Although both fractions were efficient in preventing adipocyte dysfunction after 10 days of incubation, F17 was the most efficient. It has been reported that proline, phenylalanine, and alanine (the former being found at higher concentrations in F17, and the latter found only in F17) help to prevent adipocyte dysfunction by improving glucose consumption and decreasing the production of adipokines [34, 35].

Alanine, a non-essential amino acid, can be synthesized from pyruvate and branched-chain amino acids; it is used in isonitrogenous diets for patients with obesity and comorbidities [36]. So far, no relationship of alanine with the regulation of adipogenesis has been reported, although it has been reported as related to lipolysis. Furthermore, along with L-leucine, alanine decreases the expression of fatty acid synthase, increases the expression of lipase [37], and inhibits acetyl-CoA carboxylase (ACC), thus preventing lipid accumulation in adipocytes [38]. Alanine has also been found to modify glutathione production, regulating the secretion of proinflammatory cytokines like TNF-α and IFN-γ [39]; thus, alanine could be associated with the decrease in IL-6 observed in cells treated with the F17 fraction.

On the other hand, valine, together with leucine and isoleucine, is a branched-chain amino acid (BCAA). These amino acids are essential for growth and development and are involved in the synthesis of proteins and precursors of other amino acids [40]. Valine has been reported to participate in the control of lipogenesis [33]. Since alanine can be synthesized from valine and pyruvate, and the presence of this amino acid can be related to lower IL-6 levels, the decrease in IL-6 levels induced by F18 could be attributed to its effect on the metabolism of valine, which is converted to alanine and the alanine present in the fraction. On the other hand, it has been reported that the presence of proline, phenylalanine and alanine increase the levels of adiponectin, which activates AMPK and, in turn, the absorption of glucose by adipocytes, thus regulating glucose absorption and insulin resistance [34, 35]. As for the lower IL-6 levels found in F17-treated cells, proline (which is enriched in this fraction) has shown anti-inflammatory effects by modulating the IL-6/STAT3 pathway [41]. Therefore, the involvement of alanine, valine, proline, and phenylalanine in the control of adipogenesis by PPAR-γ needs to be further evaluated.

Finally, considering that the main differences between both fractions lie in the higher proline content and the absence of phenylalanine in F18, their anti-adipogenic actions may depend on these amino acids, albeit their concentration (3.93 mM for F17 and 2.7 mM for F18), however in other studies, similar levels of amino acids have shown physiological properties [39]. It should be noted that no other compounds could be identified in these fractions, as shown in S1–S20 Figs in S1 File.

Further studies will be conducted to evaluate the effects of the amino acids and their concentrations found in the extract in their anti-adipogenic actions.

Taken together, our results indicate that the amino acids present in the effective fractions of the methanolic extract of *M*. *parviflora*, could act together to participate in the control of lipid metabolism and adipocyte differentiation. We cannot discard other molecules presented at very low levels, could act together with the found aminoacids to exert the above effects.

## 5. Conclusion

In this study, we found that fractions of *M*. *parviflora* enriched in a particular combination of alanine, valine, proline and phenylalanine for F17 or valine, proline and phenylalanine for F18 regulate adipocyte differentiation by reducing lipid accumulation, with two slightly different modes of action: F17 acts by increased AKT phosphorylation, whereas F18, while also decreasing AKT phosphorylation, significantly reduces PPAR-γ levels. It is noteworthy that both fractions inhibit adipocyte dysfunction as assessed by IL-6 production.

## Supporting information

S1 File (PDF)

S1 Raw images (PDF)
