# Supplementary material for: Anti-adipogenic effect of Malva parviflora on 3T3-L1 adipocytes
Source: PLoS One. 2024 Aug 8;19(8):e0306903. doi: 10.1371/journal.pone.0306903 (PMC11309439; doi:10.1371/journal.pone.0306903)
Supplement: S1 File — (PDF) [file pone.0306903.s001.pdf]

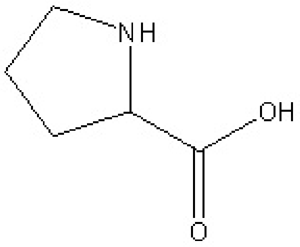

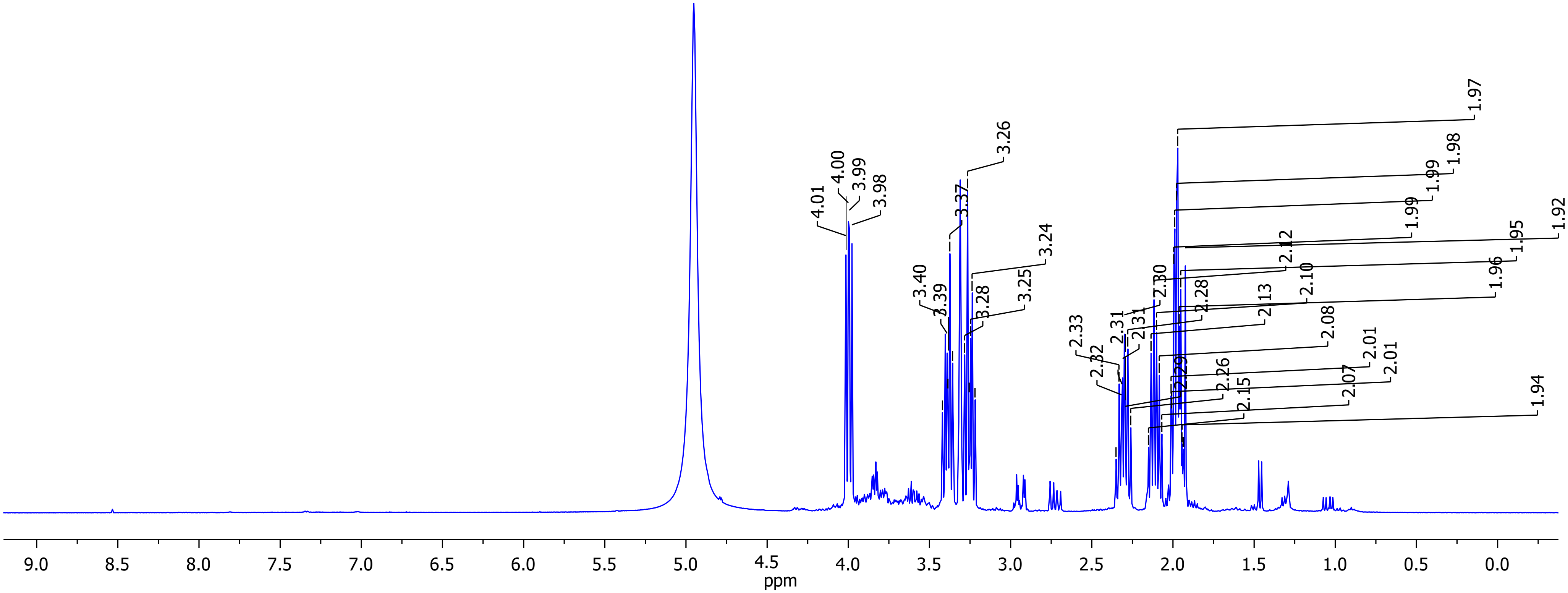

S1 Fig. ^{1}H-NMR (CD_3OD, 400 MHz) compound (**3**)

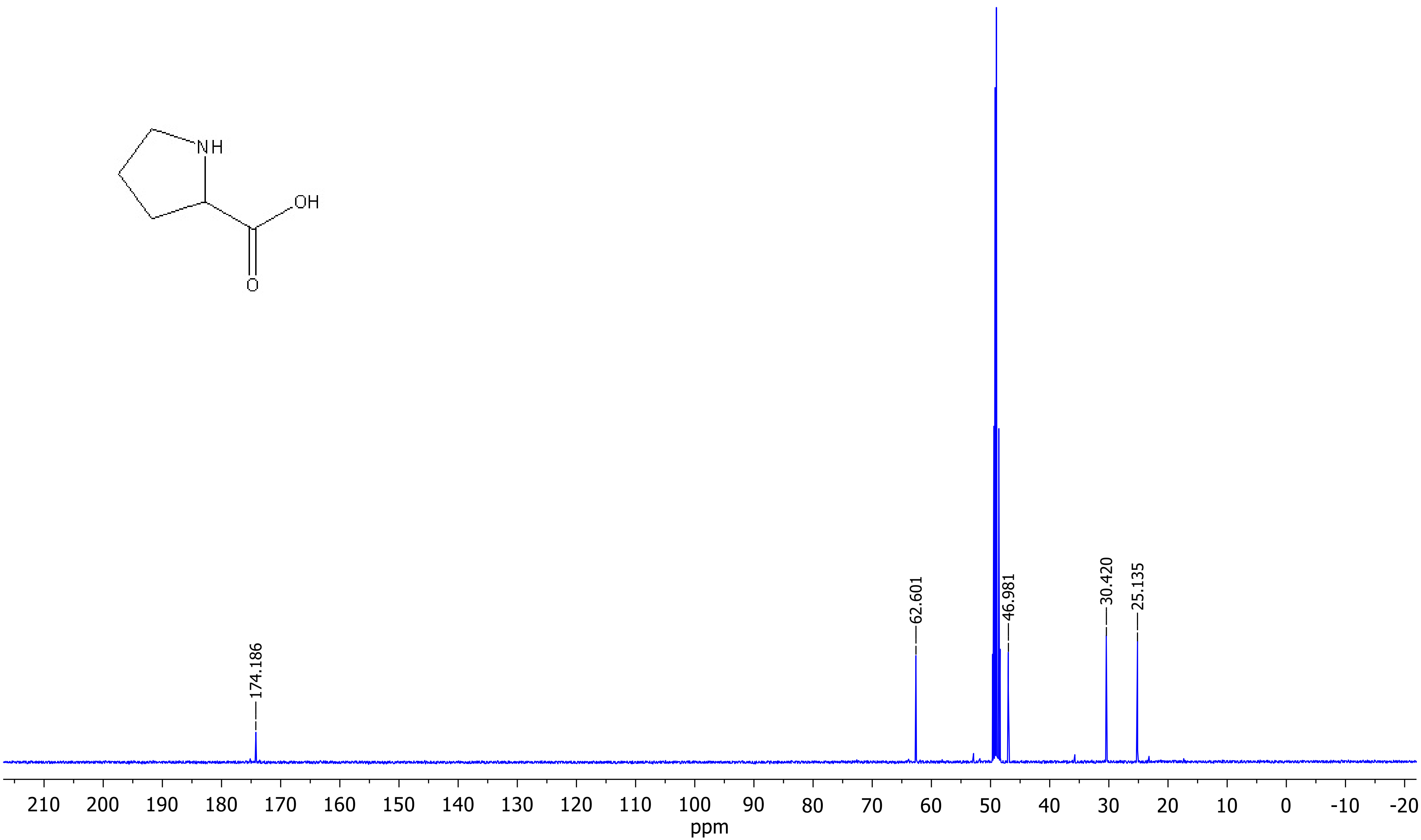

S2 Fig. ^{13}C-NMR (CD_3OD, 100 MHz) compound (**3**)

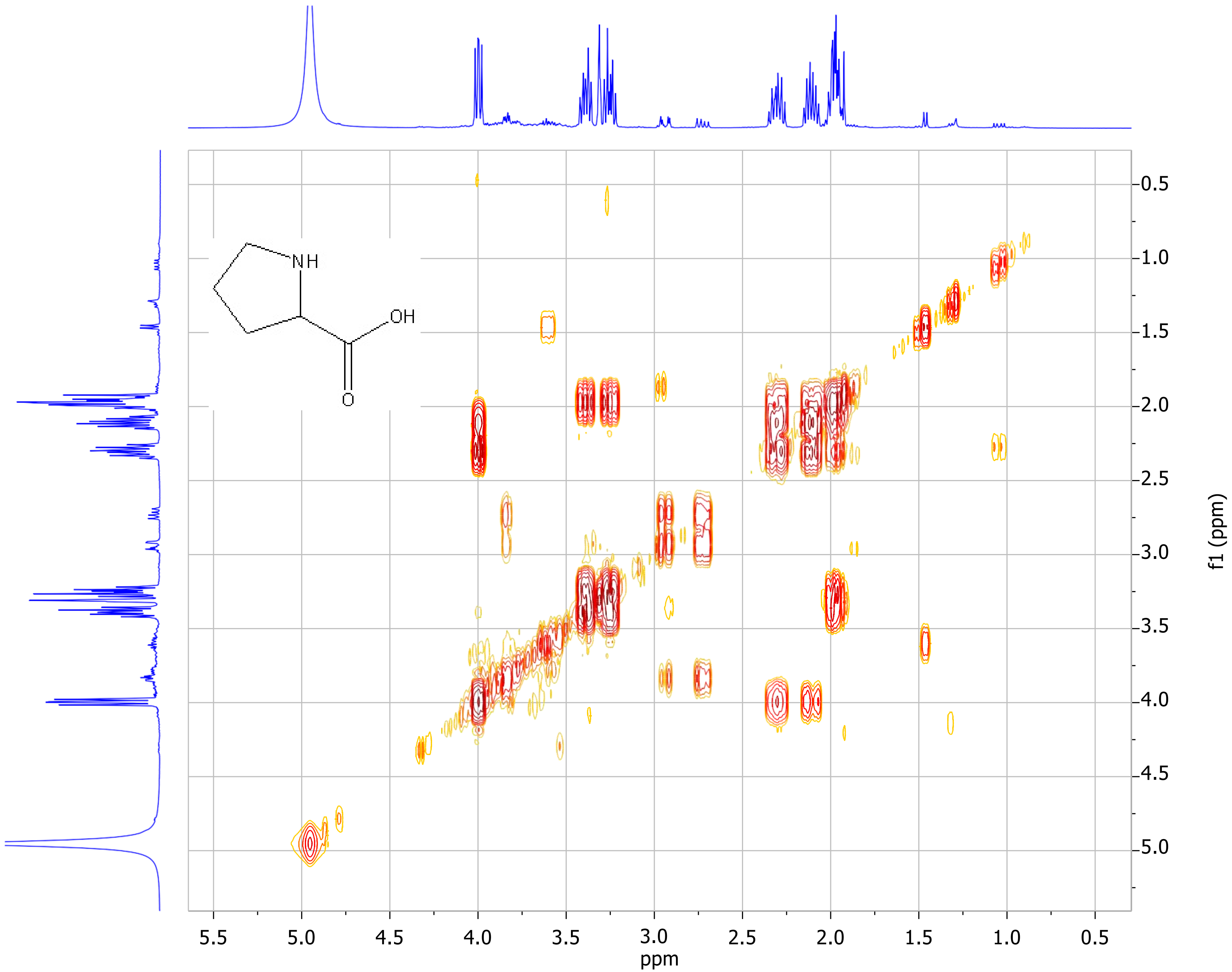

S3 Fig. ^{1}H-^{1}H(COSY)-NMR (CD_3OD, 400 MHz) compound (**3**)

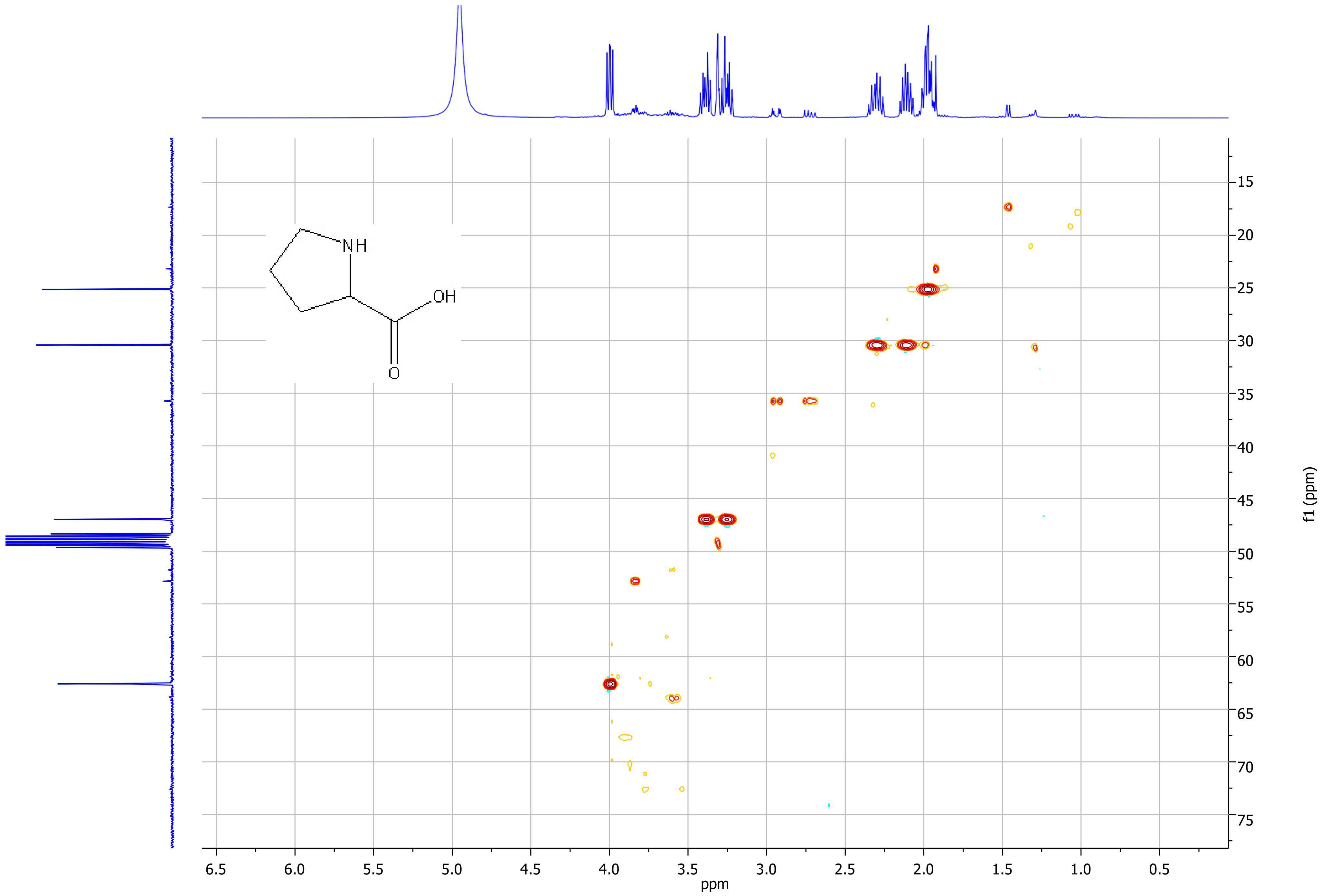

S4 Fig. ^{1}H-^{13}C(HSQC)-NMR (CD_3OD, 400 MHz) compound (**3**)

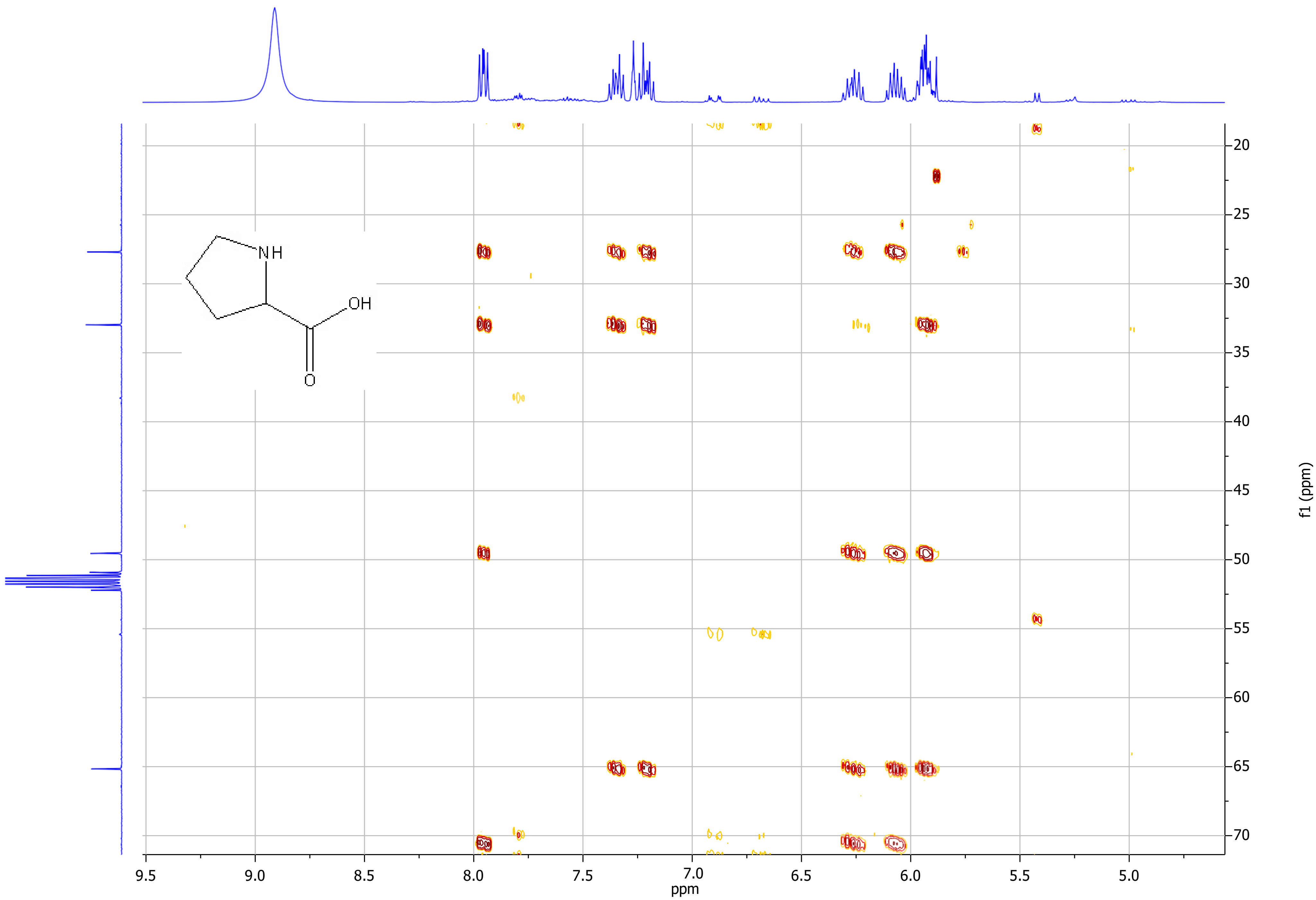

S5 Fig. ^{1}H-^{13}C(HMBC)-NMR (CD_3OD, 400 MHz) compound (**3**)

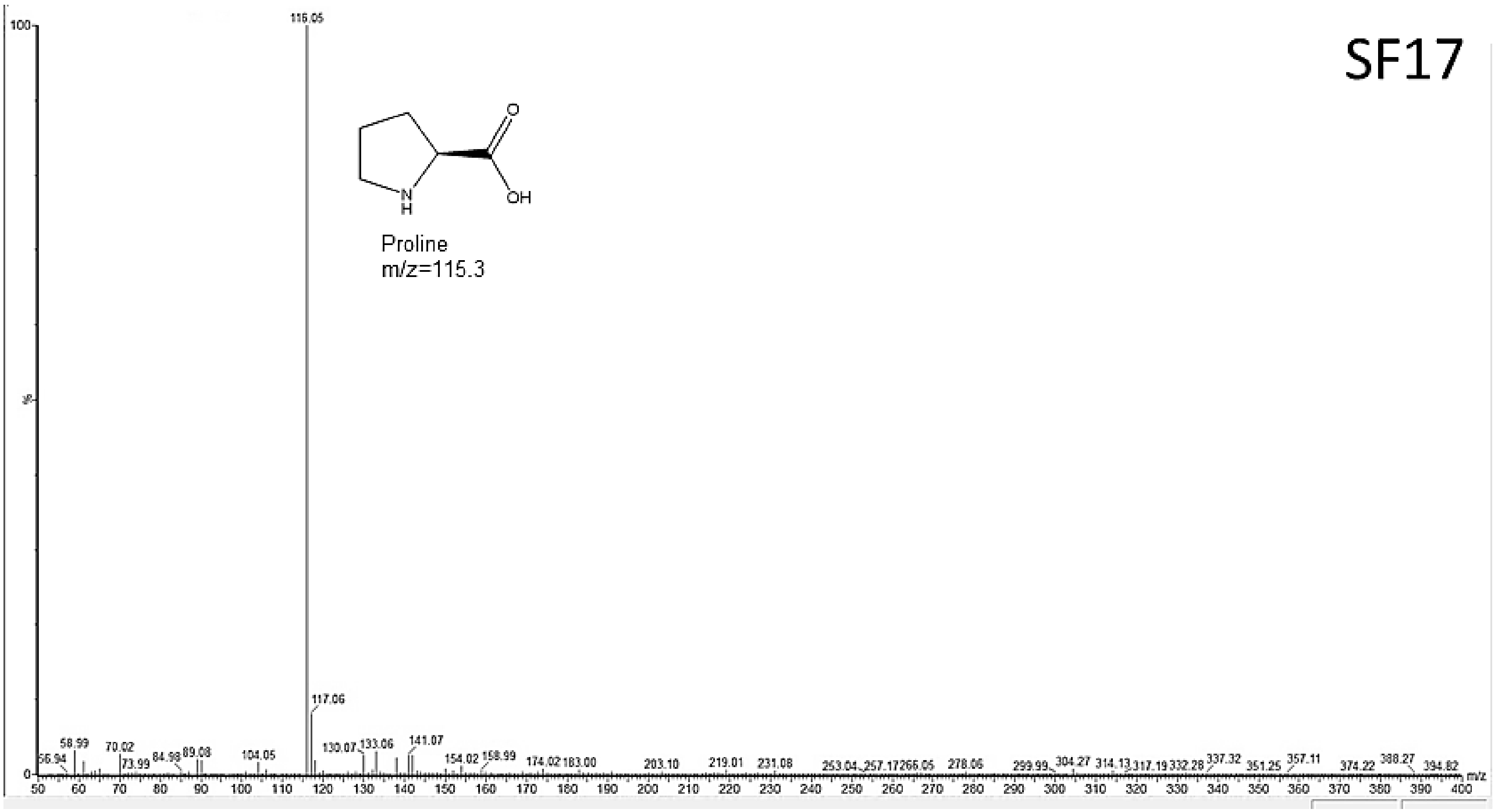

S6 Fig. Mass spectrometry SF17 compound (**3**). M+1

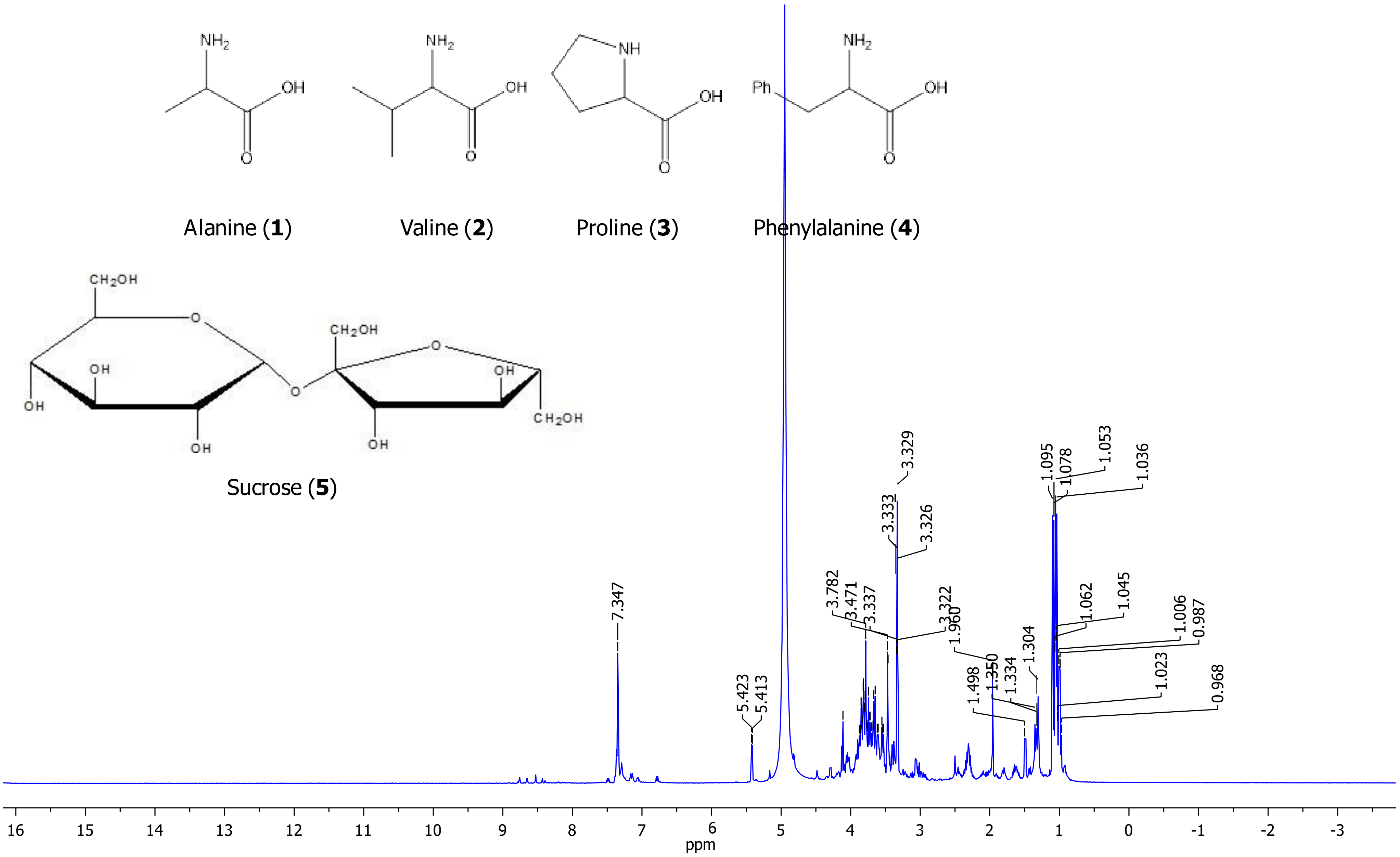

S7 Fig. ^{1}H-NMR (CD_3OD, 400 MHz) compounds (**1-5**)

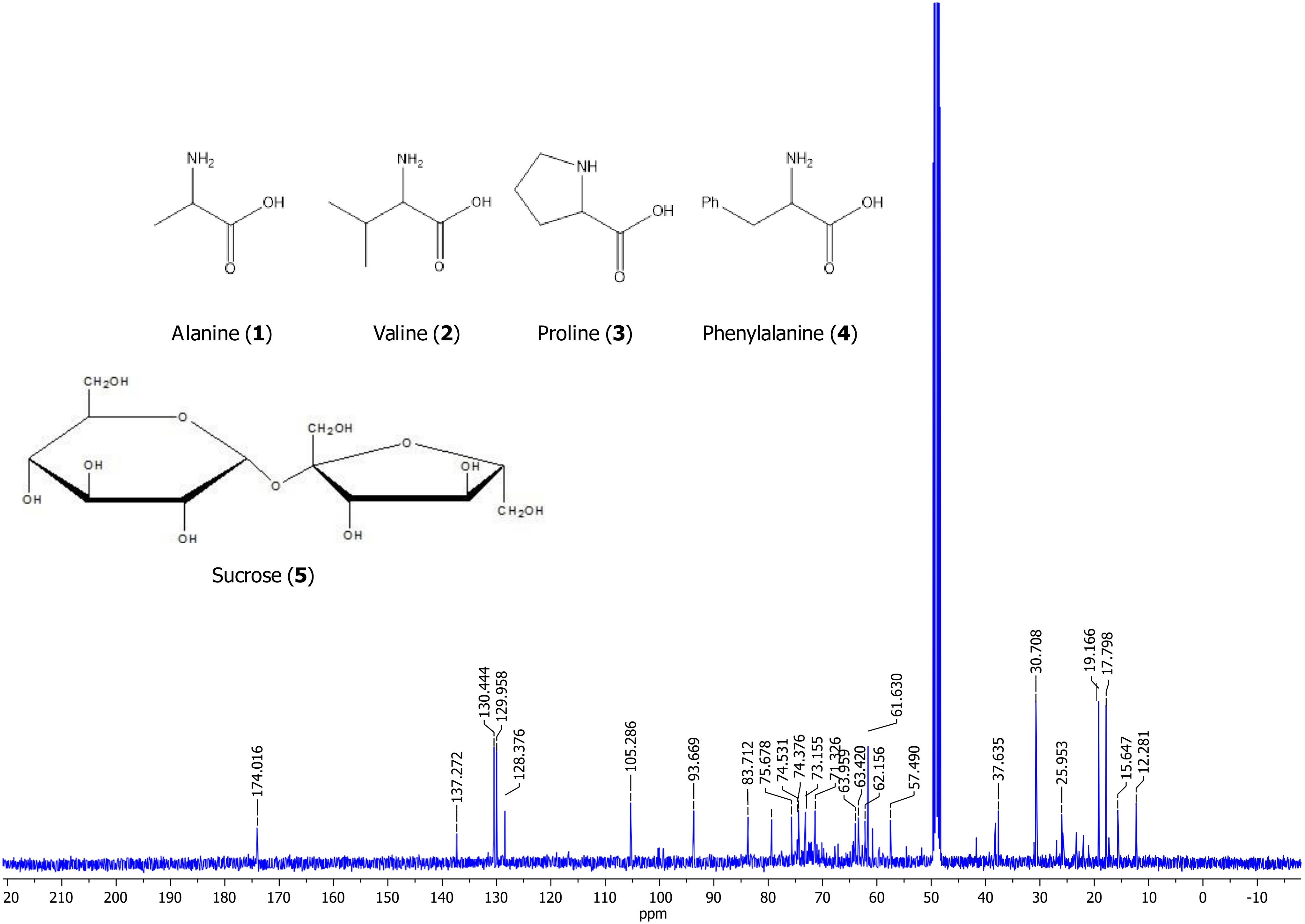

S8 Fig. ^{13}C-NMR (CD_3OD, 100 MHz) compounds (**1-5**)

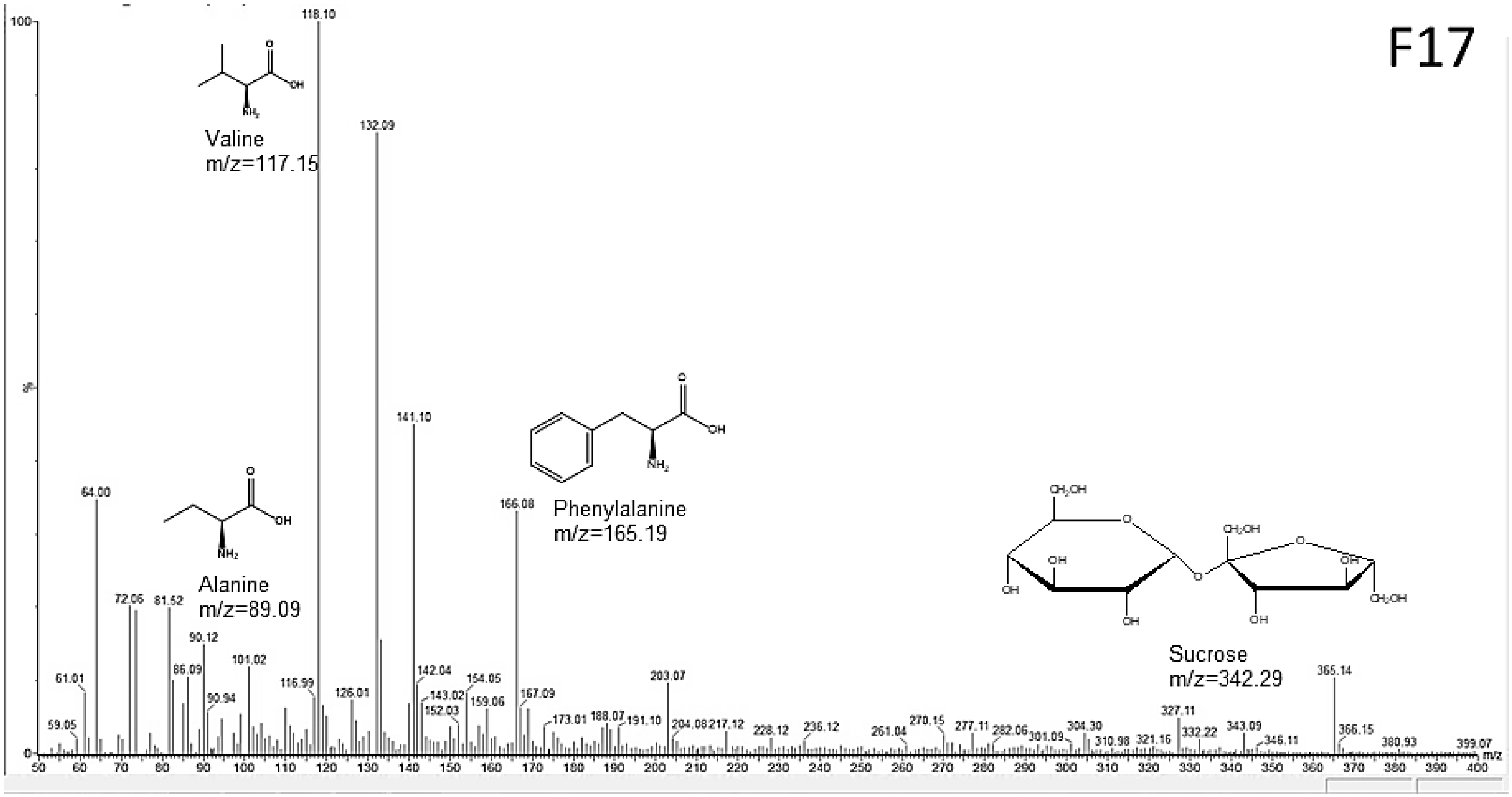

S9 Fig. Mass spectrometry F17 compound (**1, 2, 4 and 5**). M+ 1

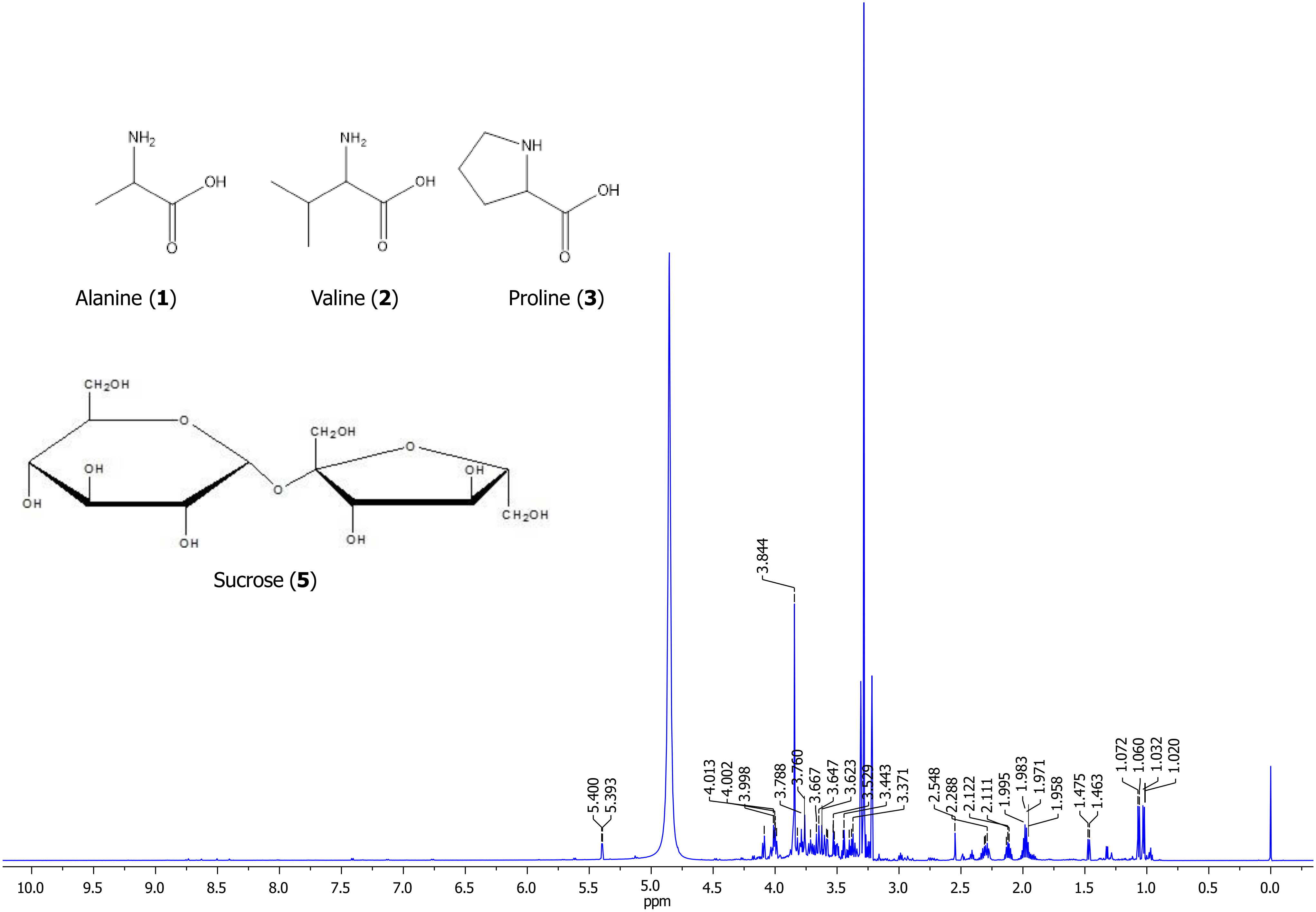

S10 Fig. ^{1}H-NMR (CD_3OD, 400 MHz) compound (**1-3, and 5**)

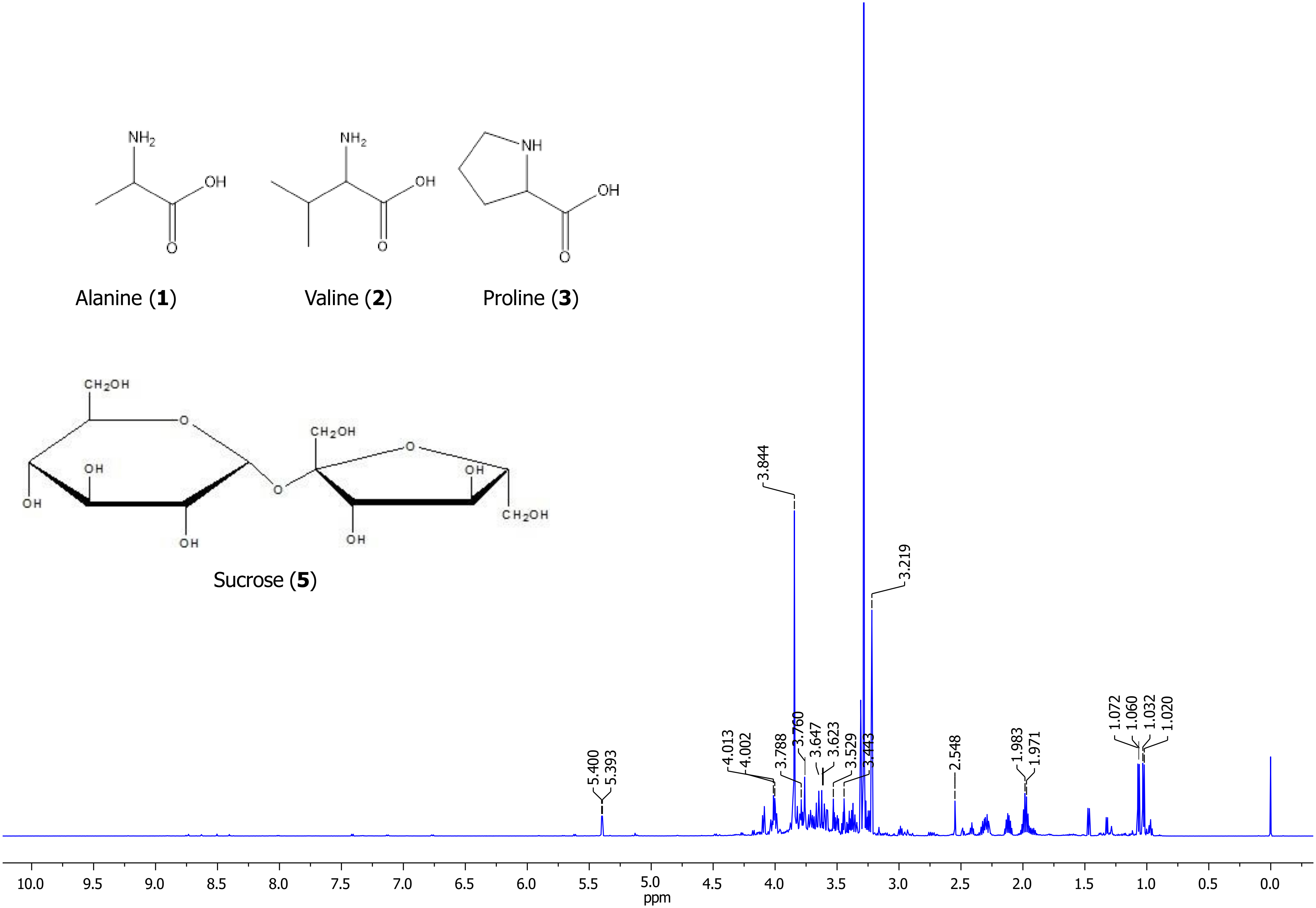

S11 Fig. [1]Hpresat-NMR (CD_3OD, 400 MHz) compounds (**1-3,5**)

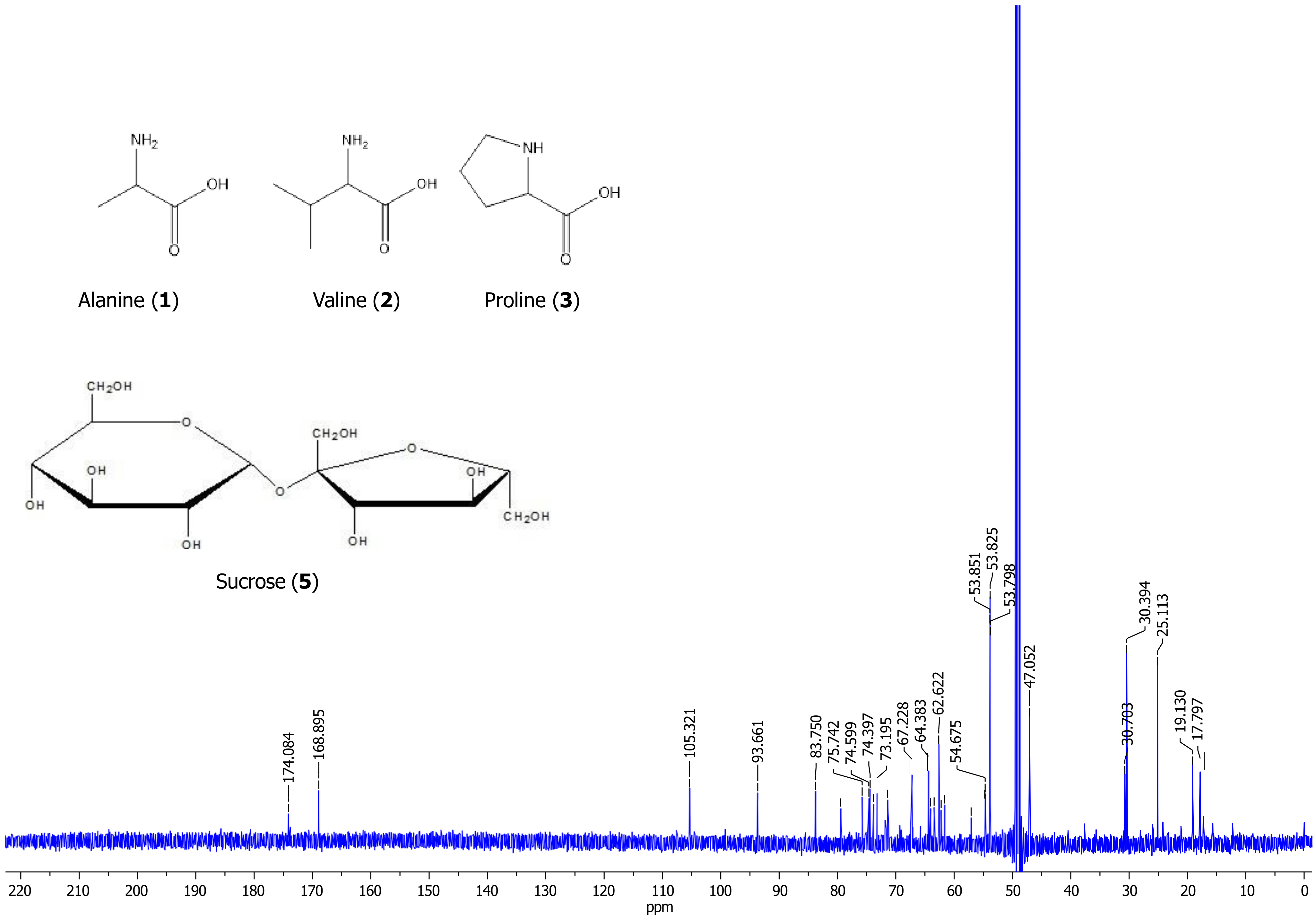

S12 Fig. ^{13}C-NMR (CD_3OD, 100 MHz) compounds (**1-3,5**)

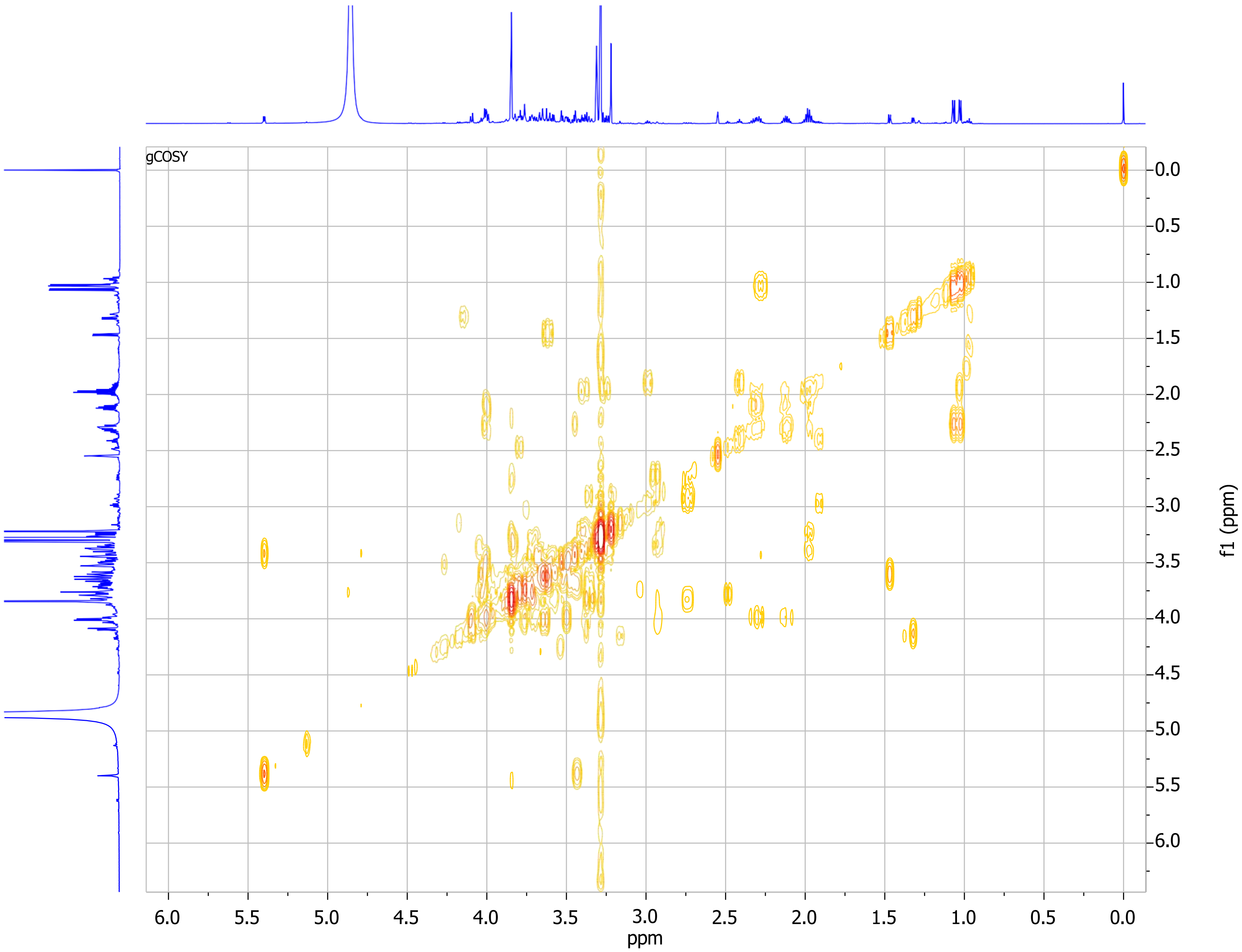

S13 Fig. ^{1}H-^{1}H(COSY)-NMR (CD_3OD, 400 MHz) compounds (**1-3,5**)

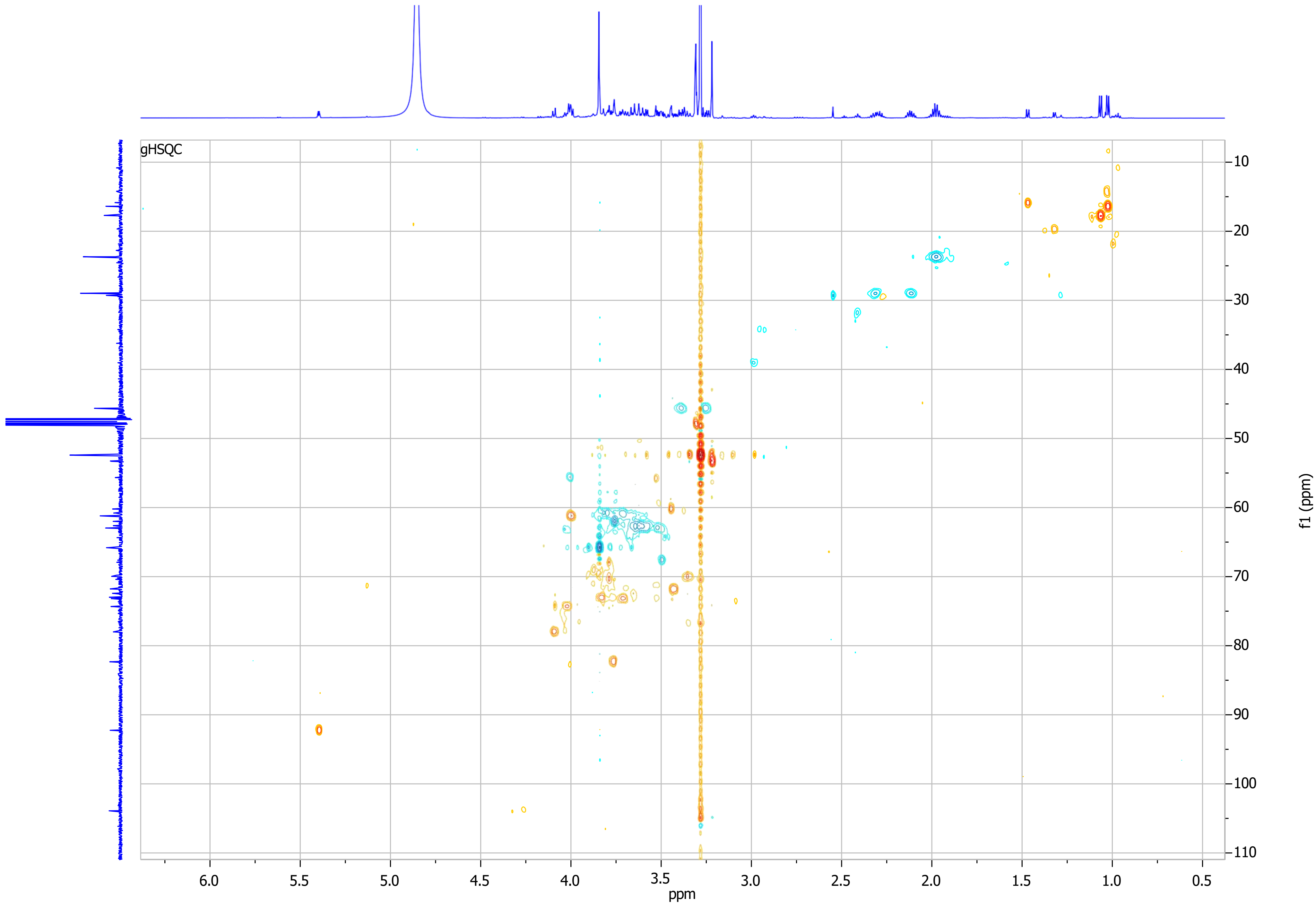

S14 Fig. ^{1}H-^{13}C(HSQC)-NMR (CD_3OD, 400 MHz) compounds (**1-3,5**)

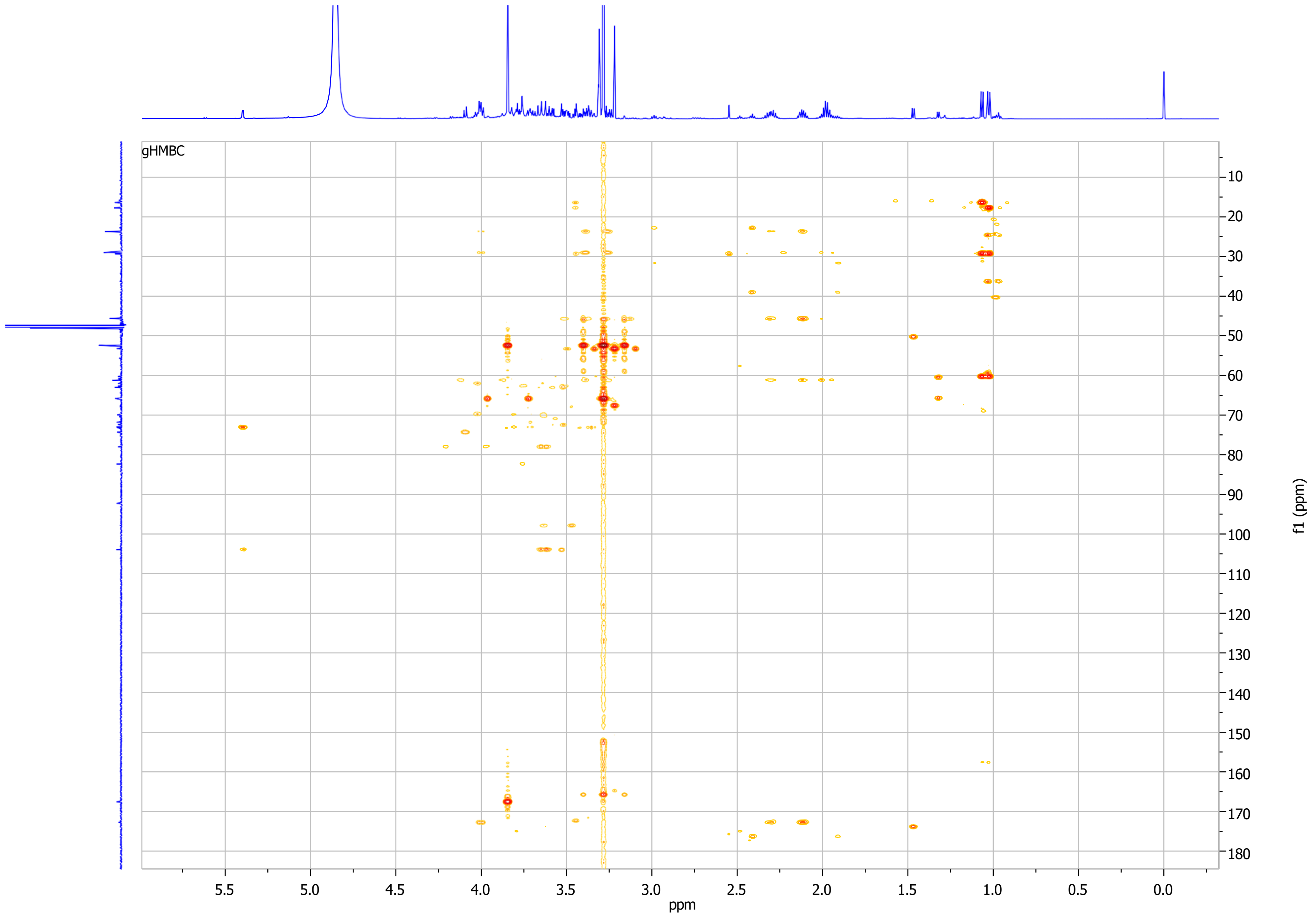

S15 Fig. ^{1}H-^{13}C(HMBC)-NMR (CD_3OD, 400 MHz) compounds (**1-3,5**)

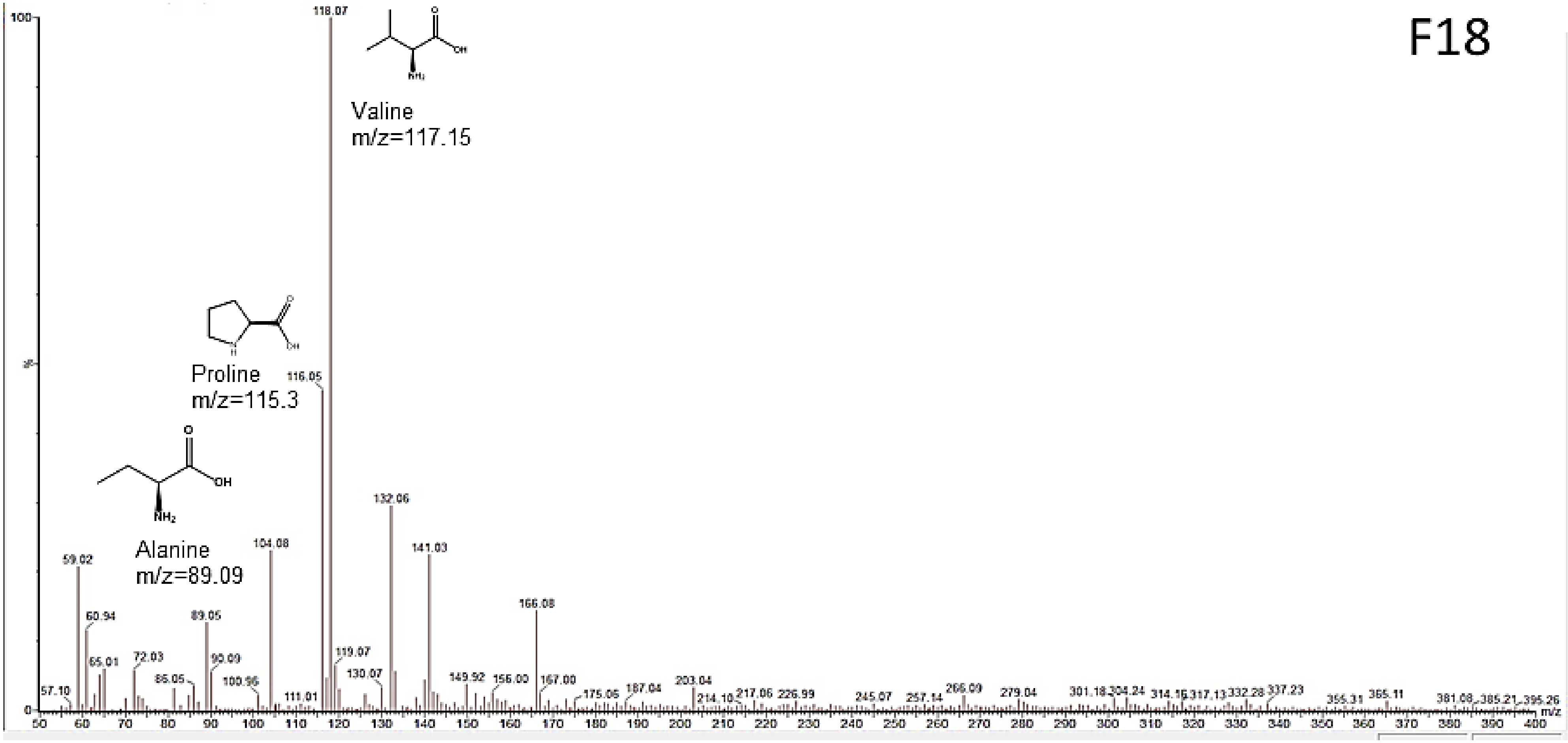

S16 Fig. Mass spectrometry F18 compound (**1,-3**). M+1

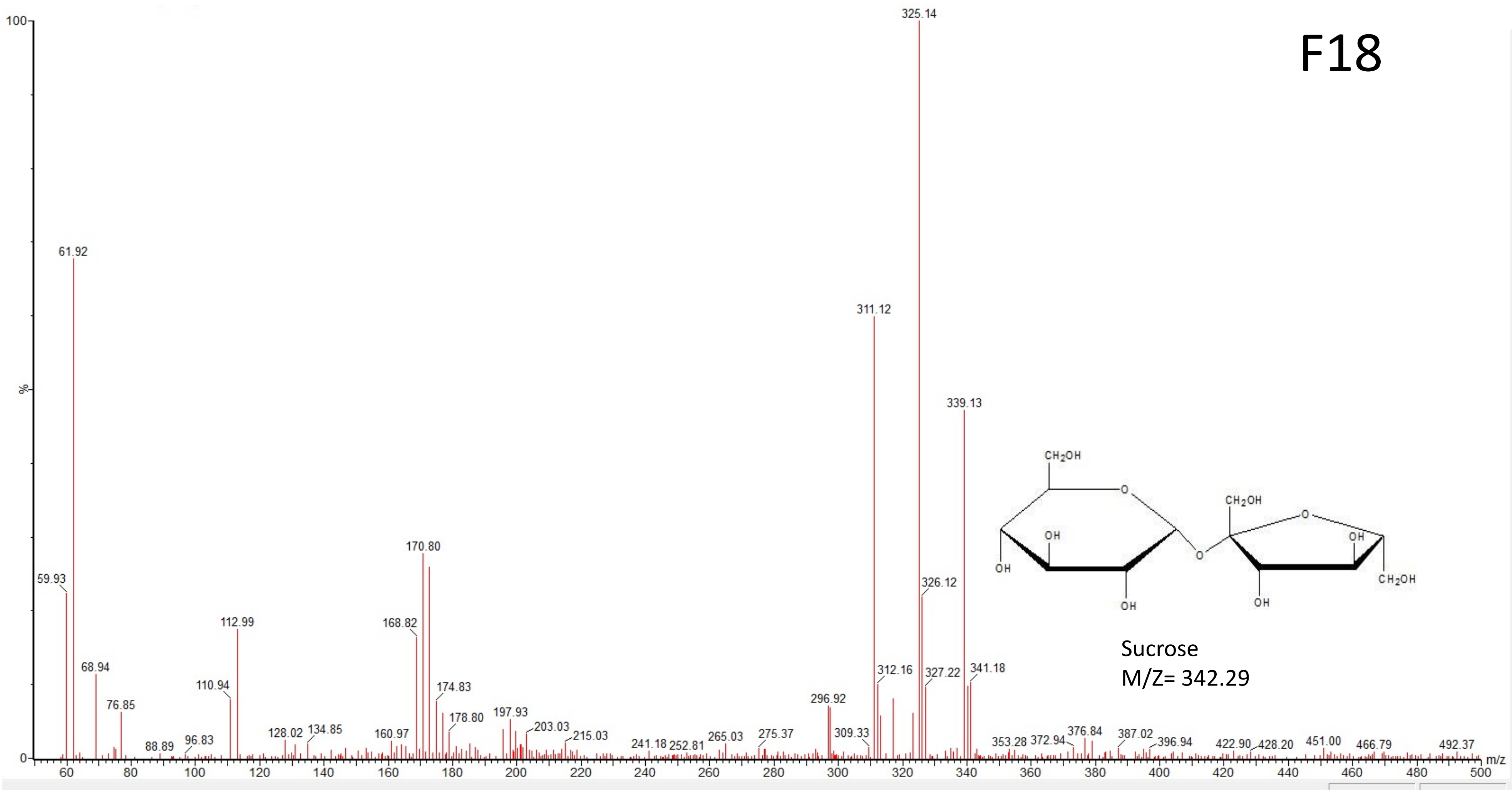

S17 Fig. Mass spectrometry F18 compound (**5**). M-1

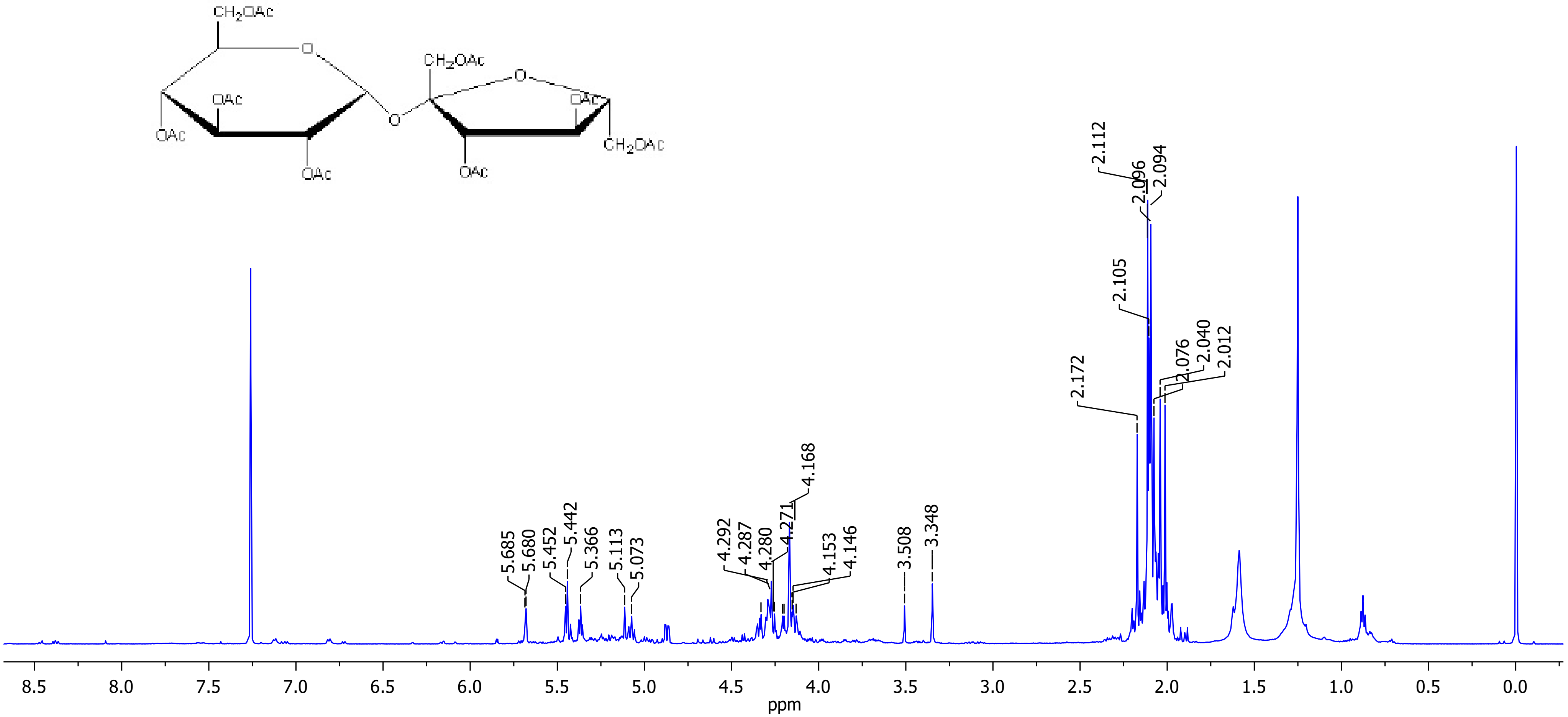

S18 Fig. ^{1}H-NMR ($CDCl_3$, 400 MHz) acetylated fracction F18 (Sucrose peracetylated)

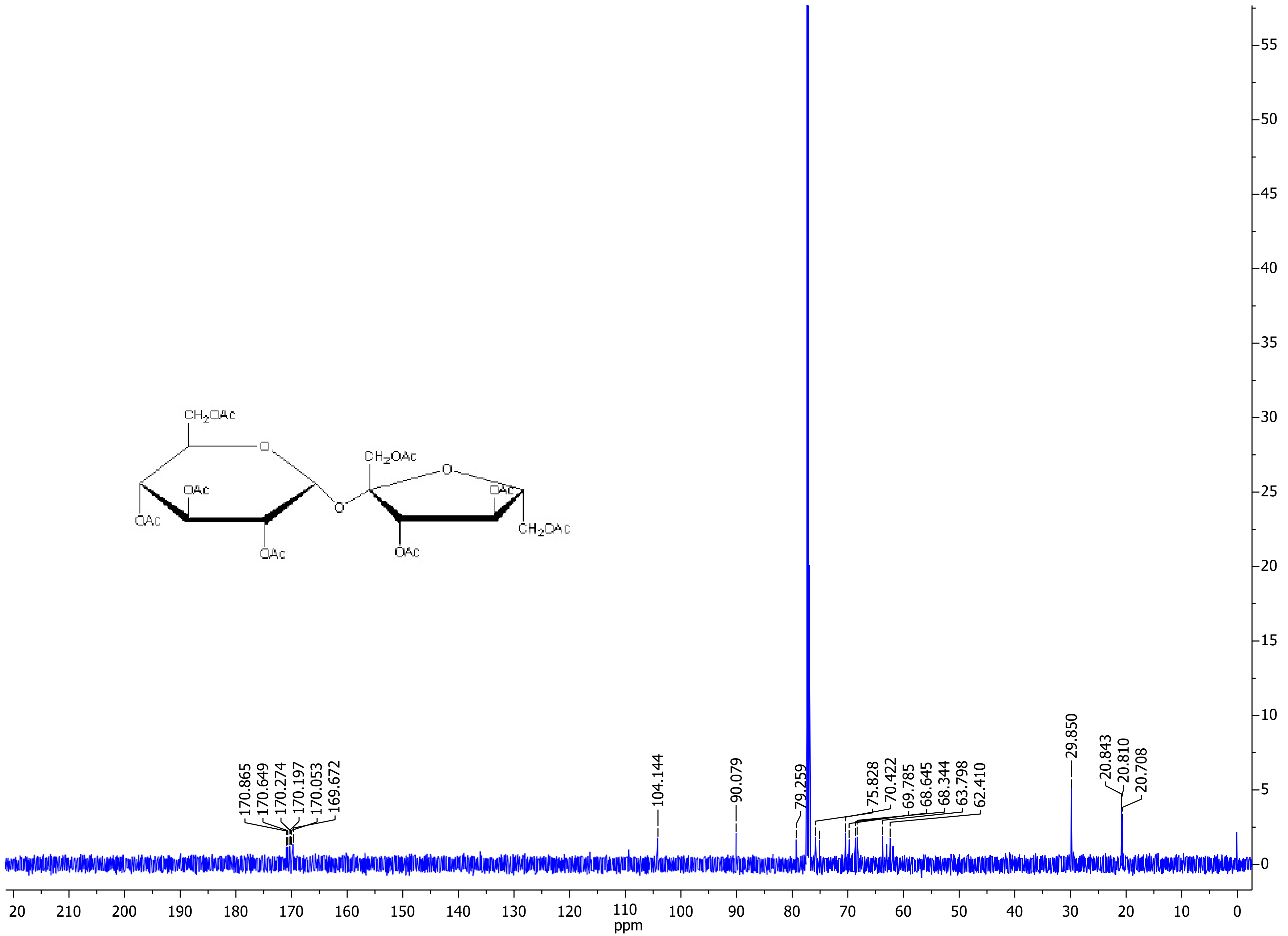

S19 Fig. ^{13}C-NMR ($CDCl_3$, 100 MHz) acetylated fracction F18 (Sucrose peracetylated)

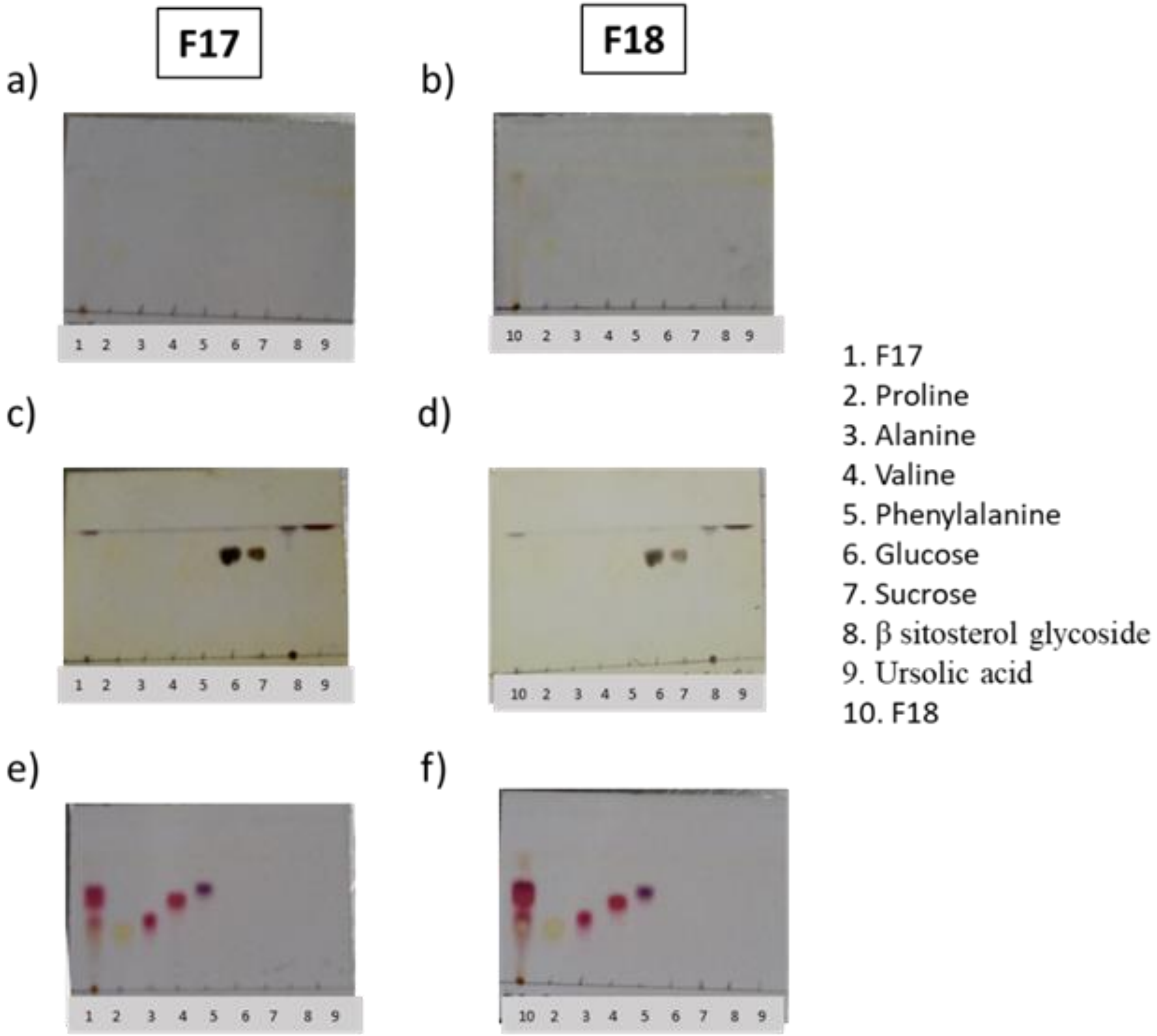

S20 Fig. Thin-layer chromatography. Separation of compounds in the fractions of *Malva parviflora* (F17 and F18) using normal-phase TLC and the system ethyl acetate:methanol:water:glacial acetic acid (5:5:2:0.5 v/v/v/v). the spots were sprayed whit Vanillin-H_2SO_4 (a-b), ceric sulfate (c-d) and ninhydrin (e-f).

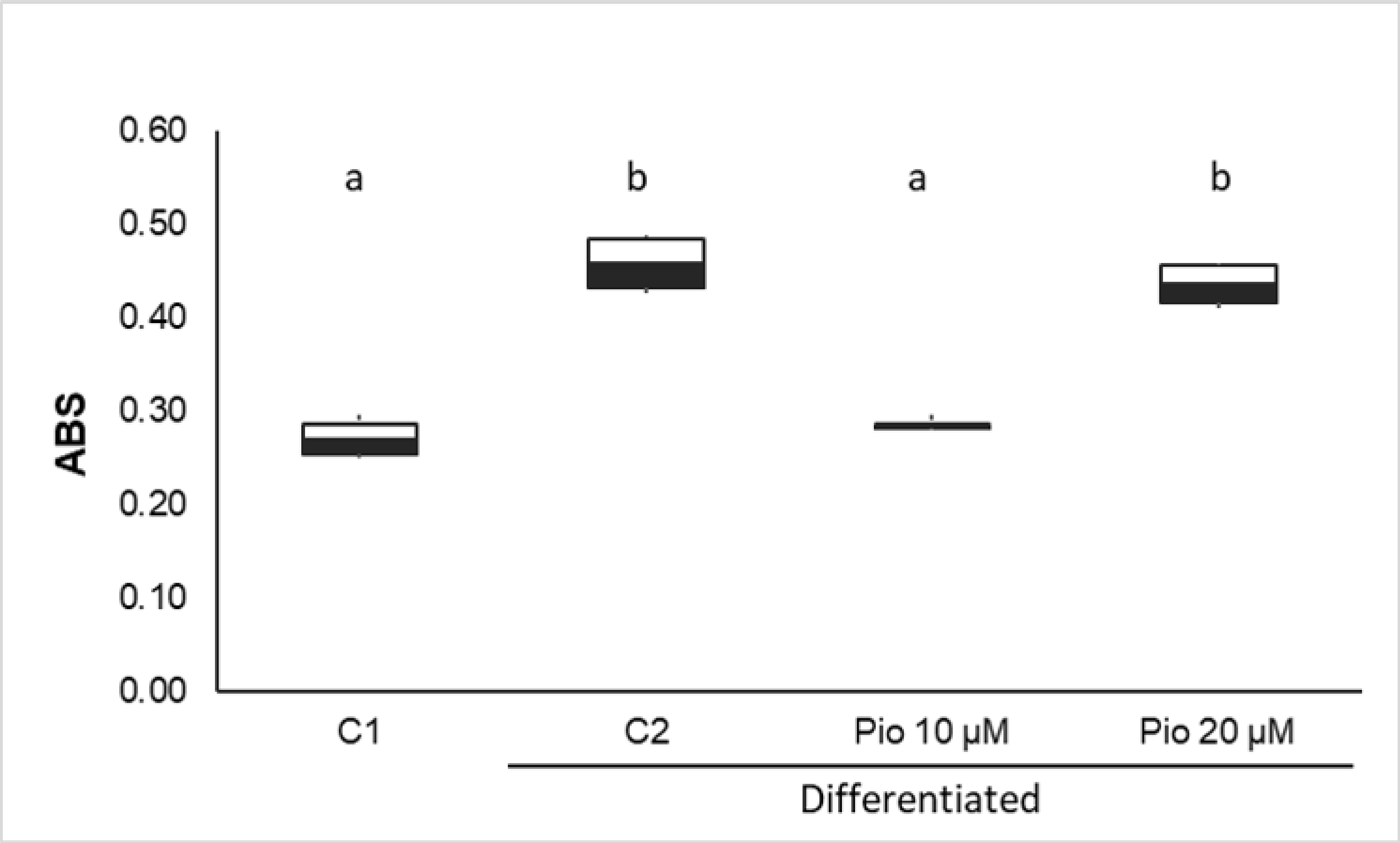

S21 Fig. Effect for pioglitazone the lipogenesis.
