# Supplementary material for: Anti-adipogenic effect of Malva parviflora on 3T3-L1 adipocytes
Source: PLoS One. 2024 Aug 8;19(8):e0306903. doi: 10.1371/journal.pone.0306903 (PMC11309439; doi:10.1371/journal.pone.0306903)

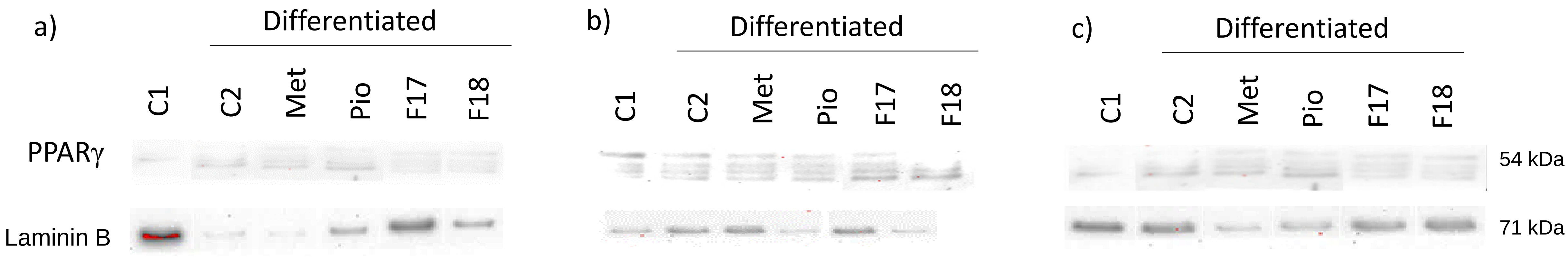

C1: control cells
C2: differentiated cells
Met: Metformin 10 mM
Pio: Pioglitazone 20 µg/mL
F17: Fraction 17 of *M. parviflora* 14 µg/mL
F18: Fraction 18 of *M. parviflora* 17 µg/mL

The bands were analyzed using the software ImageJ 1.8.0
The panel b was used to make fig 3b

a) PPARγ

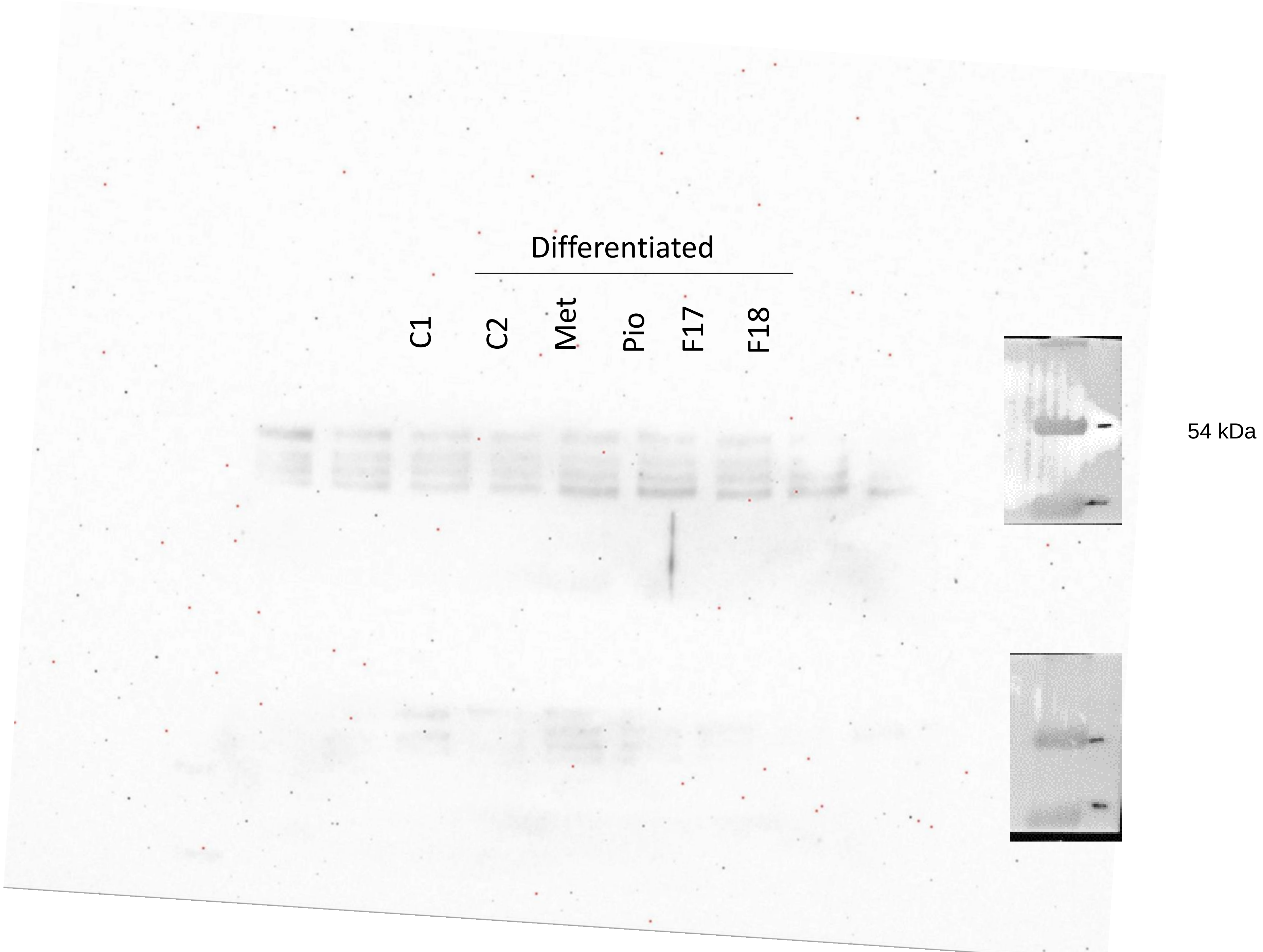

b) PPARγ

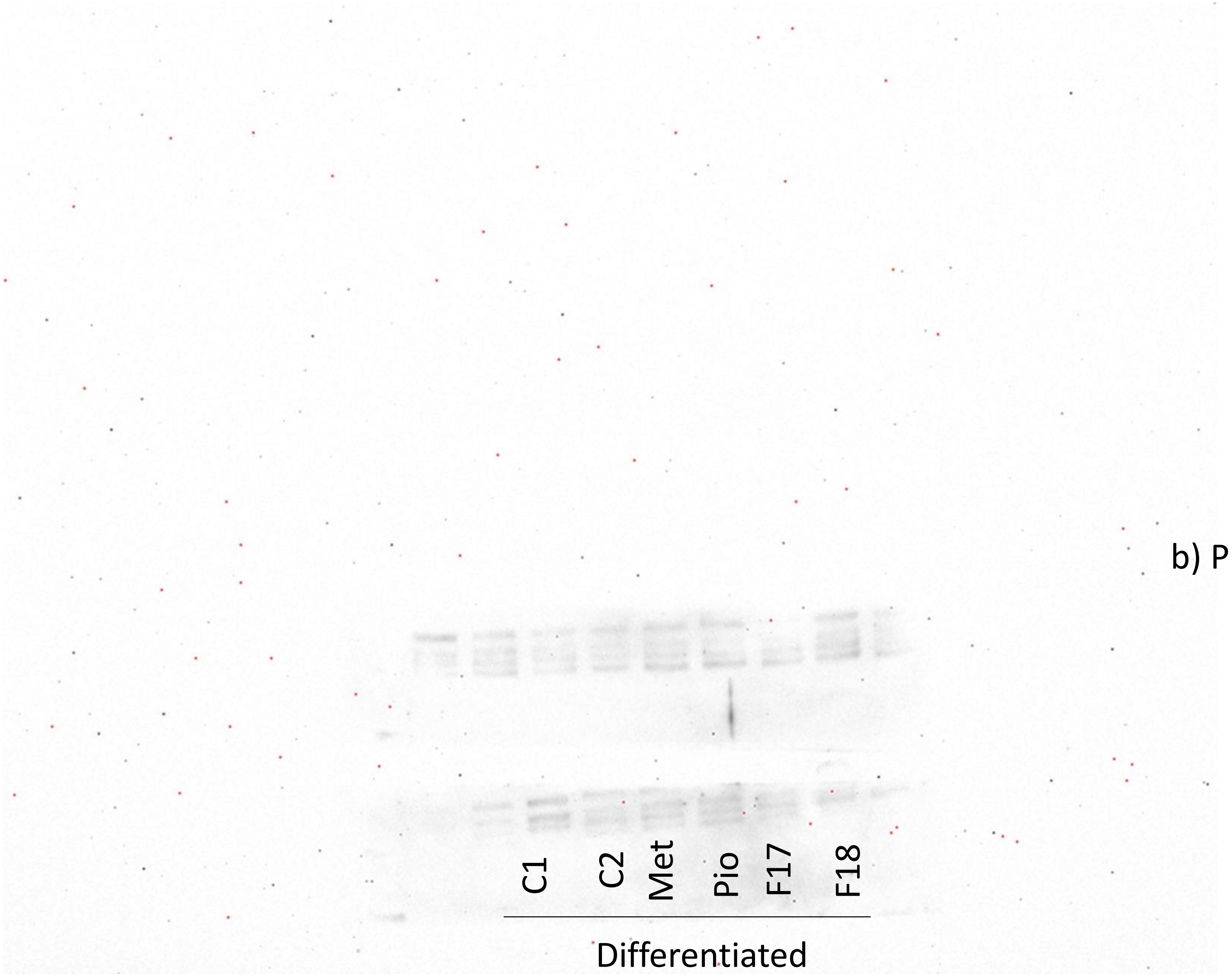

c) PPARγ

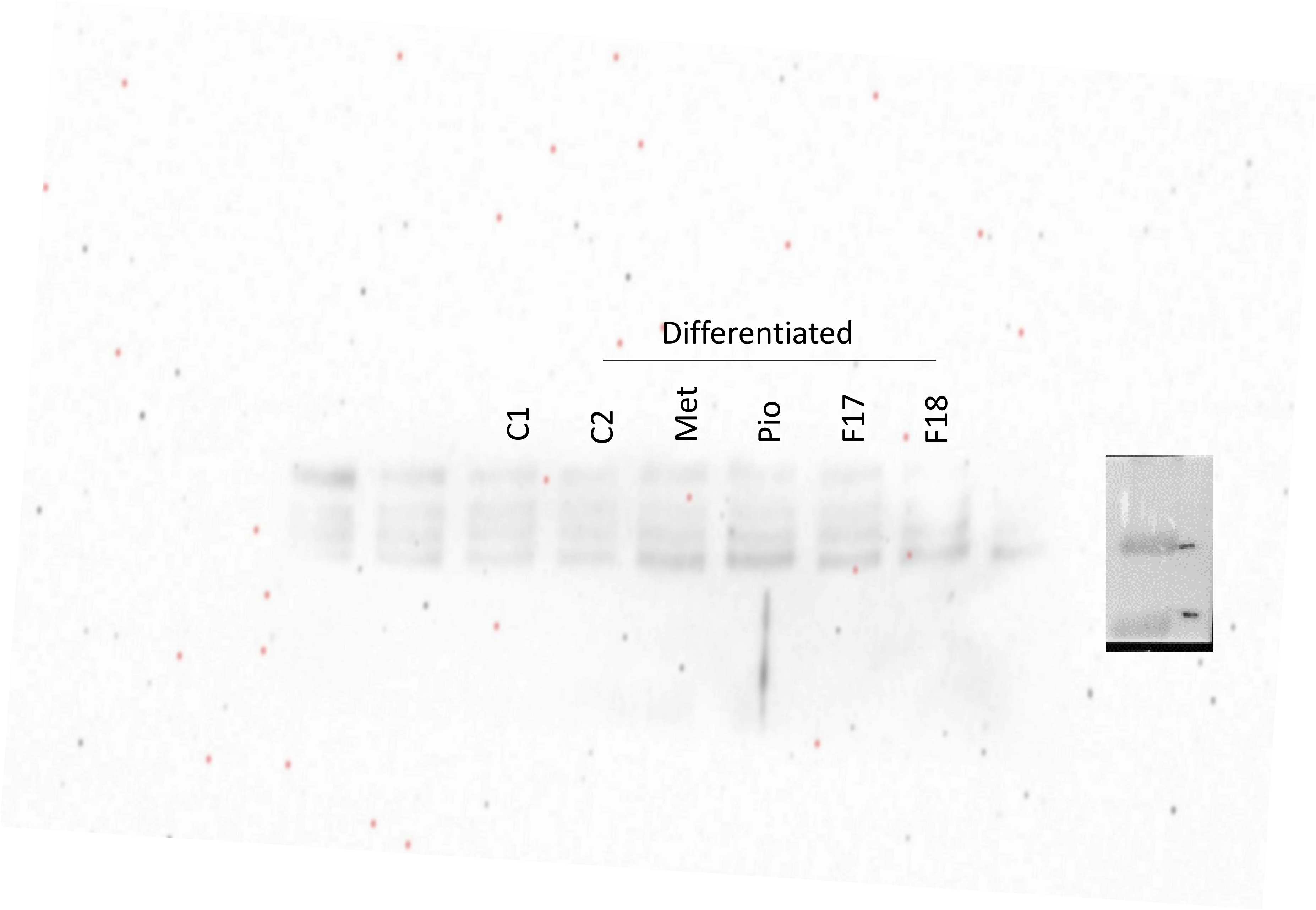

c) Laminin

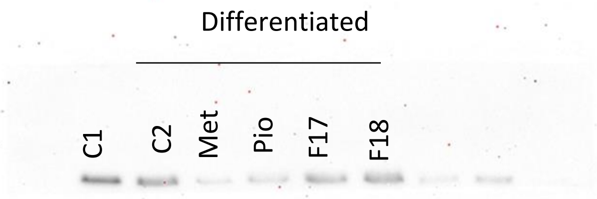

a) Laminin

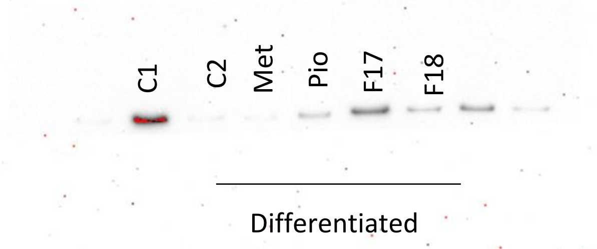

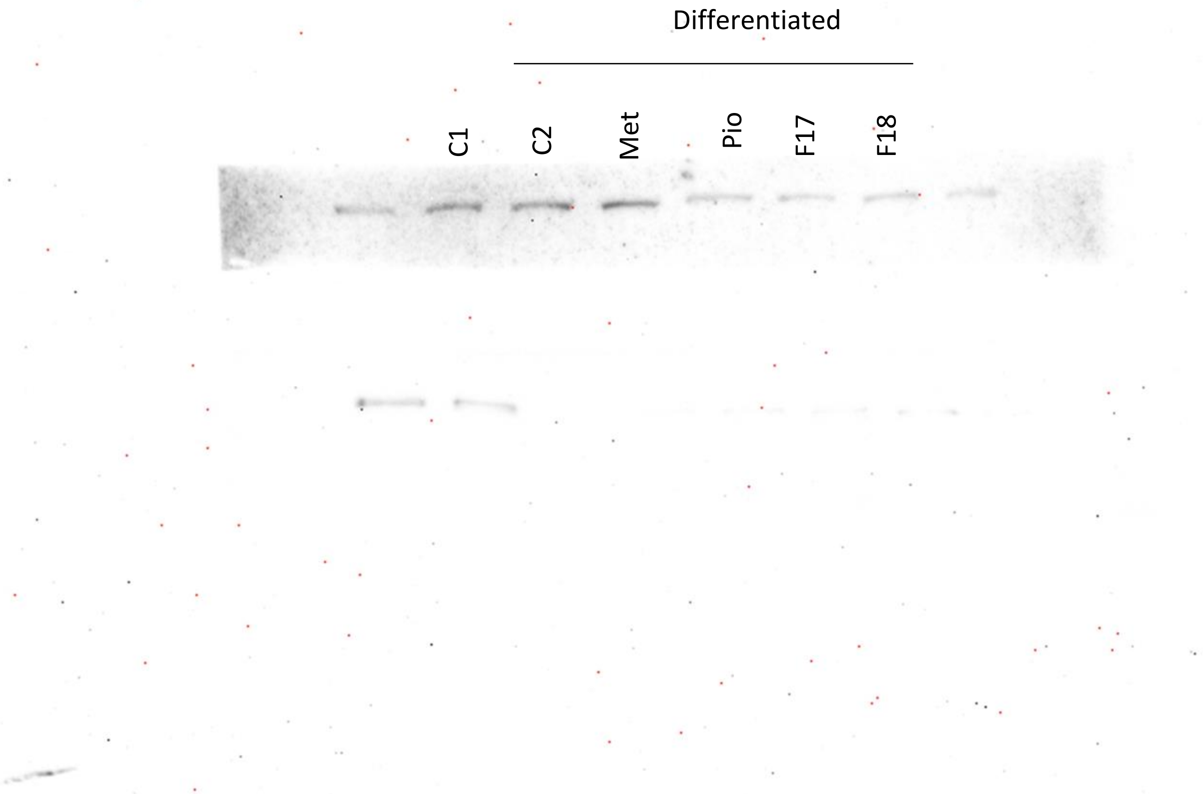

b) Laminin

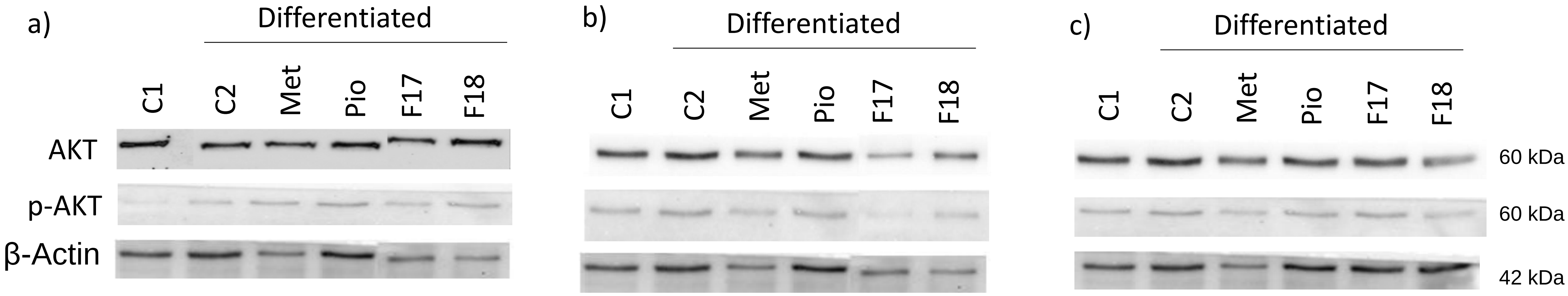

C1: control cells
C2: differentiated cells
Met: Metformin 10 mM
Pio: Pioglitazone 20 µg/mL
F17: Fraction 17 of *M. parviflora* 14 µg/mL
F18: Fraction 18 of *M. parviflora* 17 µg/mL

The bands were analyzed using the software ImageJ 1.8.0
The panel b was used to make fig 4a

a) AKT

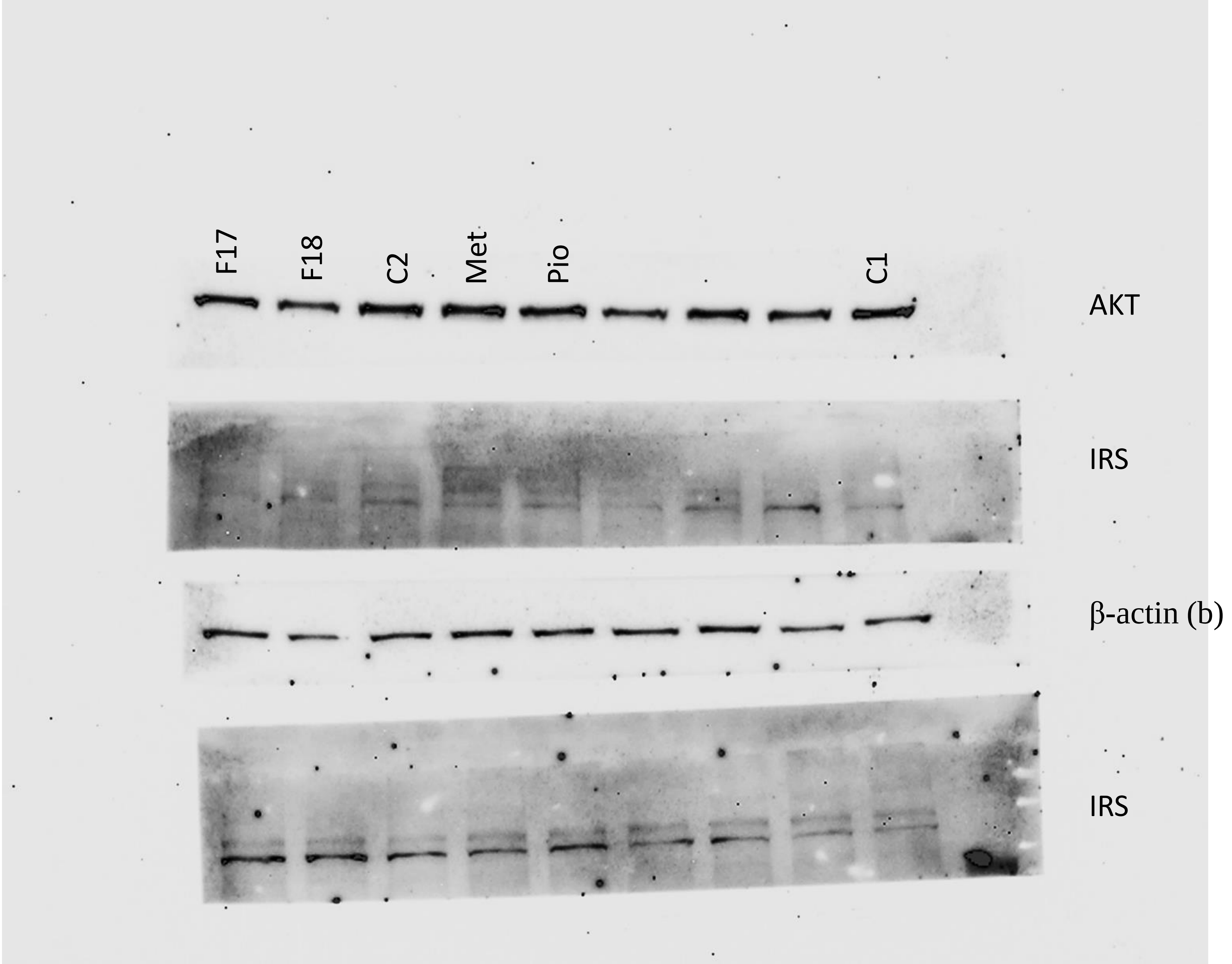

b) AKT

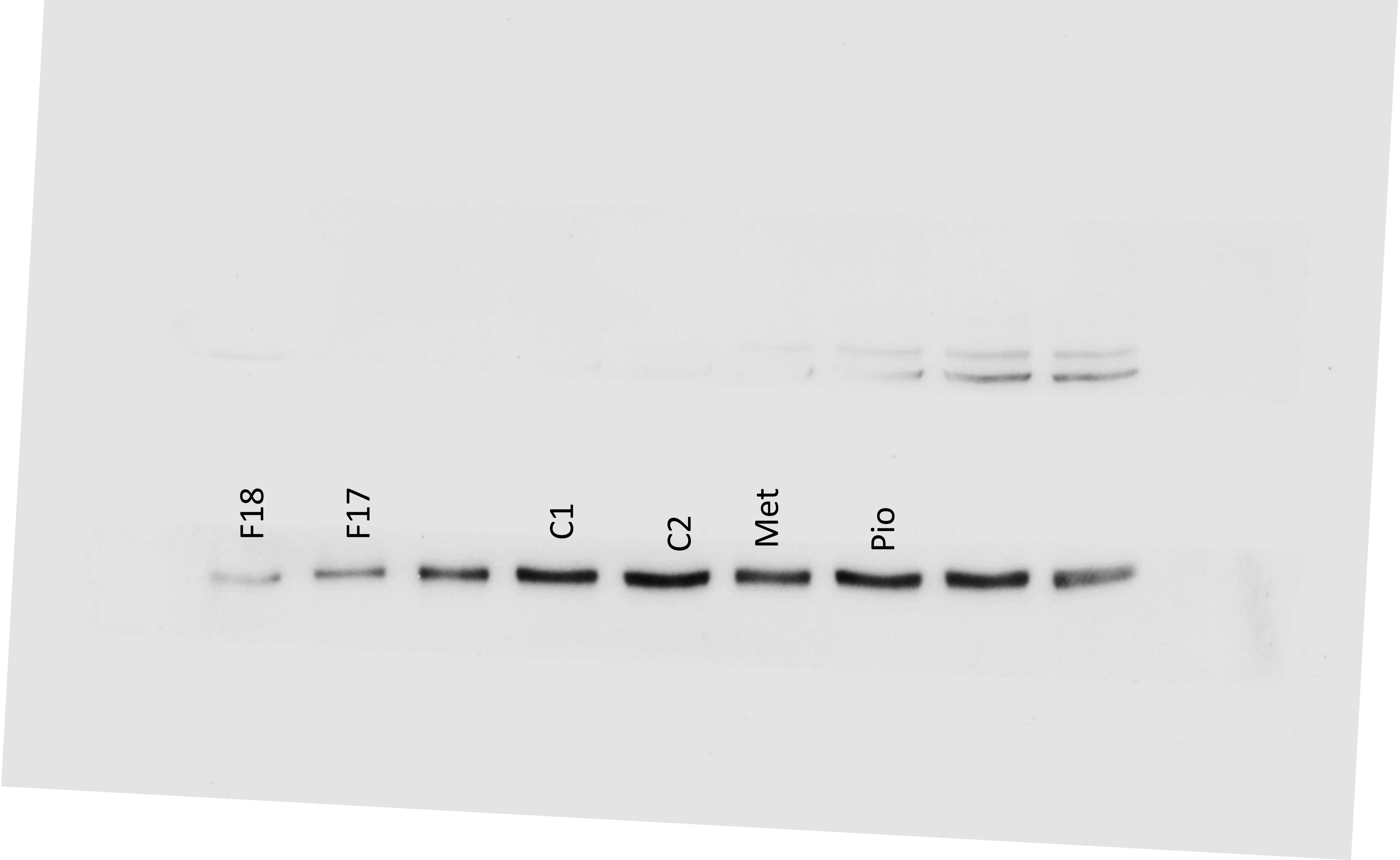

c) AKT

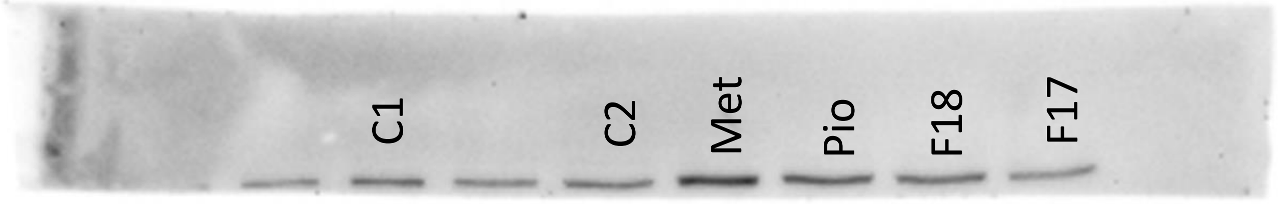

a) p-AKT

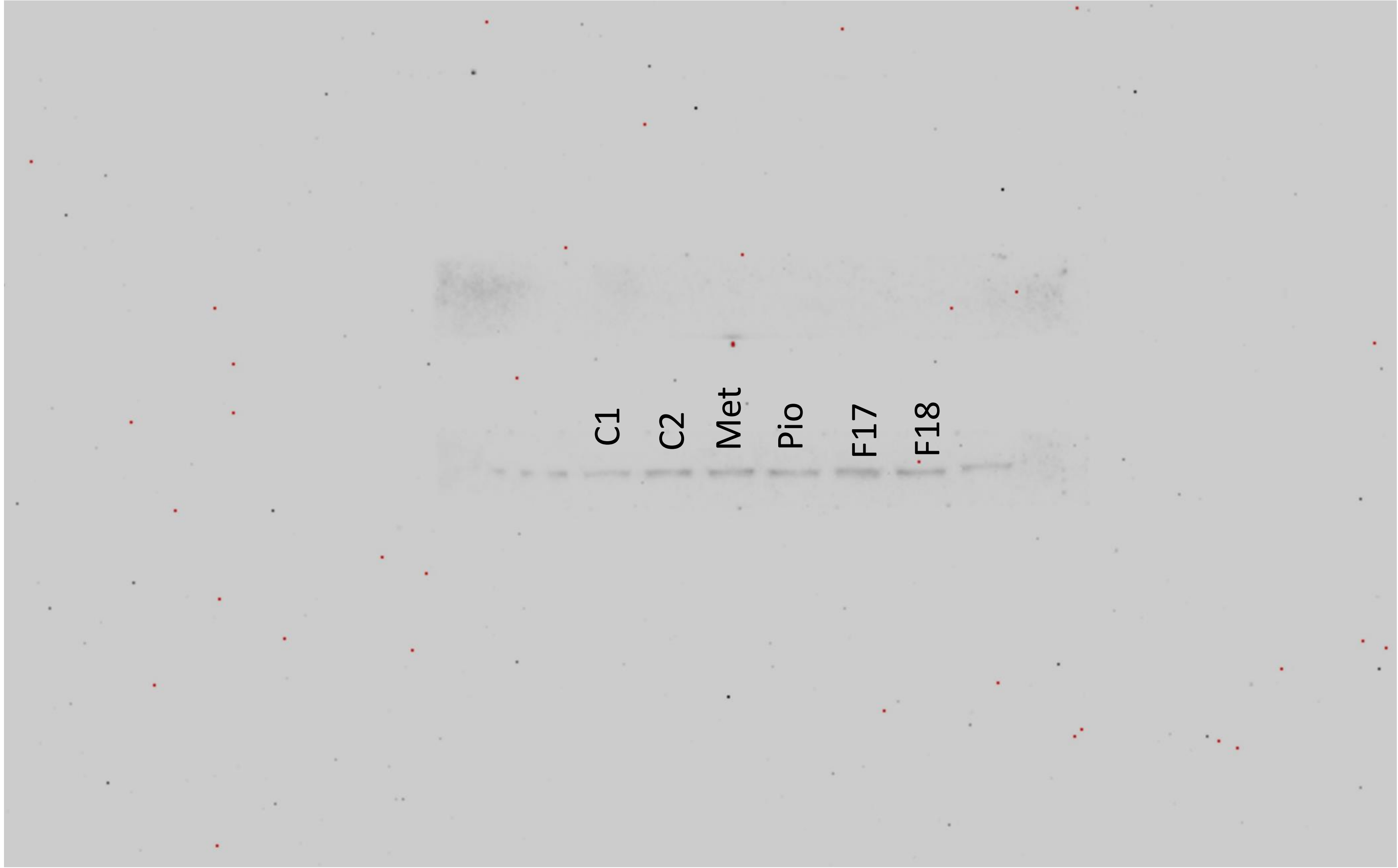

b) p-AKT

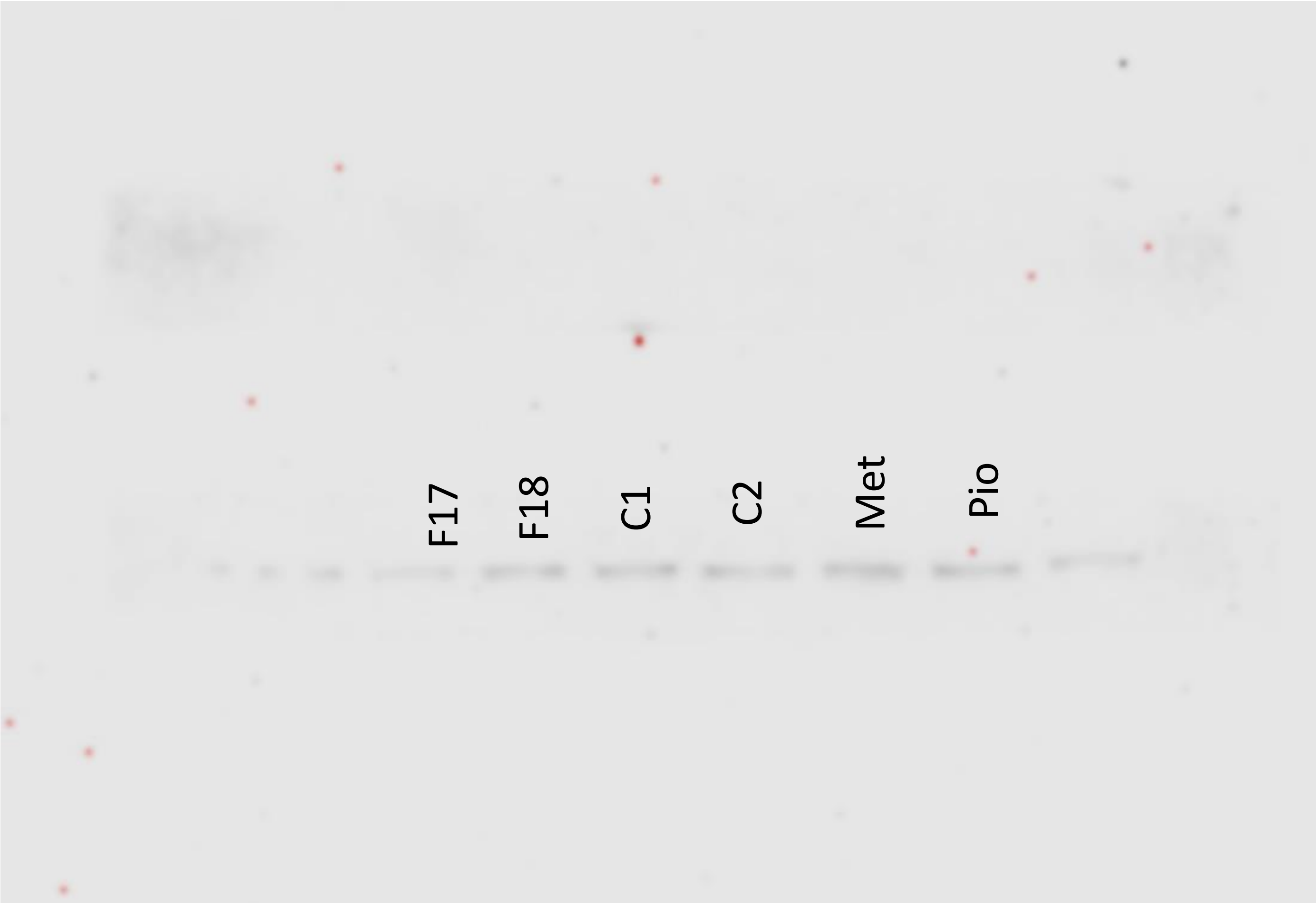

c) p-AKT

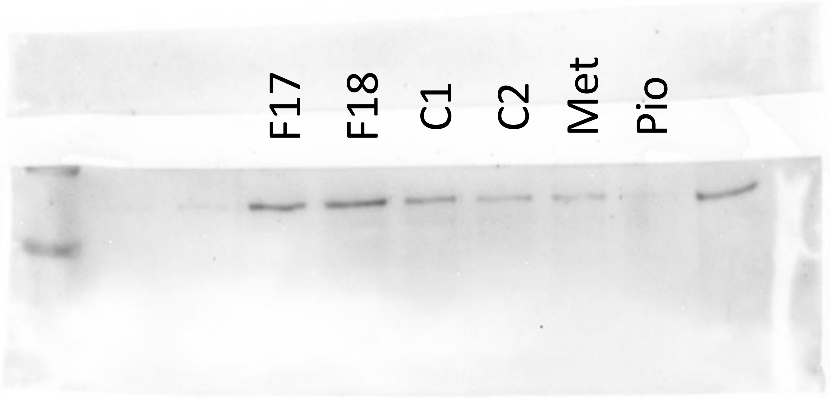

b) β-actin

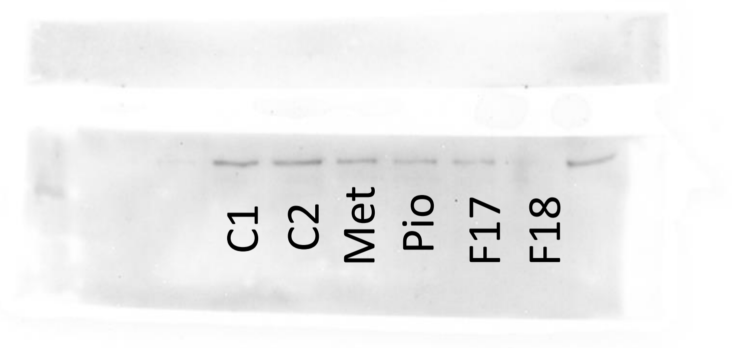

c) β-actin

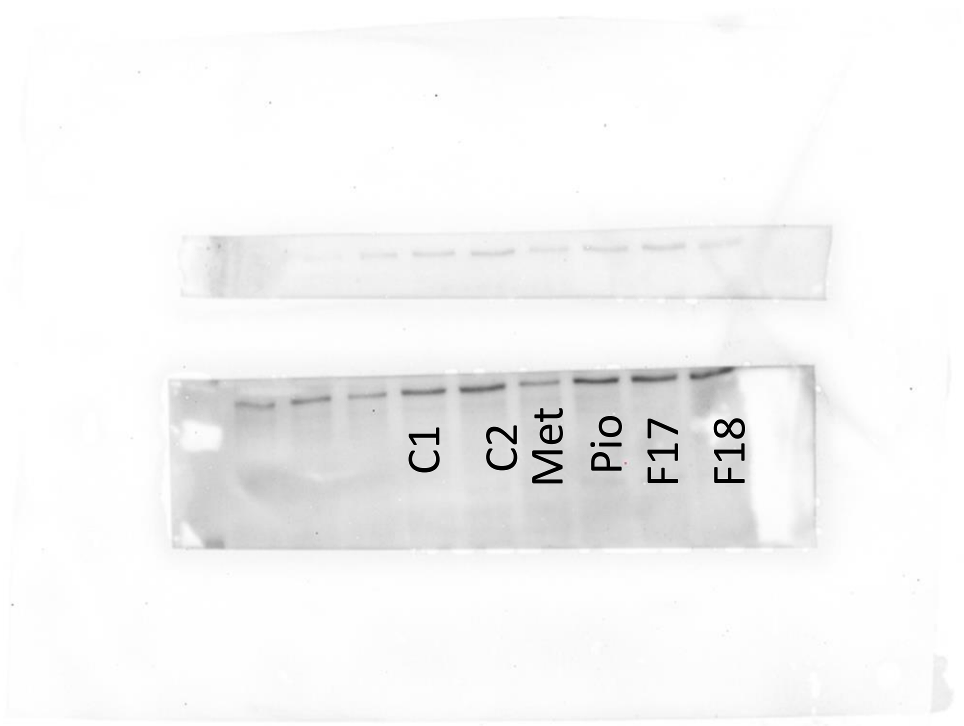

Supplement: S1 Raw images — (PDF) [file pone.0306903.s002.pdf]
